# Significant Increase in Ecosystem C Can Be Achieved with Sustainable Forest Management in Subtropical Plantation Forests

**DOI:** 10.1371/journal.pone.0089688

**Published:** 2014-02-24

**Authors:** Xiaohua Wei, Juan A. Blanco

**Affiliations:** 1 Department of Earth and Environmental Sciences, University of British Columbia, Kelowna, British Columbia, Canada; 2 Departmento de Ciencias del Medio Natural, Universidad Pública de Navarra, Pamplona, Navarra, Spain; Tennessee State University, United States of America

## Abstract

Subtropical planted forests are rapidly expanding. They are traditionally managed for intensive, short-term goals that often lead to long-term yield decline and reduced carbon sequestration capacity. Here we show how it is possible to increase and sustain carbon stored in subtropical forest plantations if management is switched towards more sustainable forestry. We first conducted a literature review to explore possible management factors that contribute to the potentials in ecosystem C in tropical and subtropical plantations. We found that broadleaves plantations have significantly higher ecosystem C than conifer plantations. In addition, ecosystem C increases with plantation age, and reaches a peak with intermediate stand densities of 1500–2500 trees ha^−1^. We then used the FORECAST model to simulate the regional implications of switching from traditional to sustainable management regimes, using Chinese fir (*Cunninghamia lanceolata*) plantations in subtropical China as a study case. We randomly simulated 200 traditional short-rotation pure stands and 200 sustainably-managed mixed Chinese fir – *Phoebe bournei* plantations, for 120 years. Our results showed that mixed, sustainably-managed plantations have on average 67.5% more ecosystem C than traditional pure conifer plantations. If all pure plantations were gradually transformed into mixed plantations during the next 10 years, carbon stocks could rise in 2050 by 260.22 TgC in east-central China. Assuming similar differences for temperate and boreal plantations, if sustainable forestry practices were applied to all new forest plantation types in China, stored carbon could increase by 1,482.80 TgC in 2050. Such an increase would be equivalent to a yearly sequestration rate of 40.08 TgC yr^−1^, offsetting 1.9% of China’s annual emissions in 2010. More importantly, this C increase can be sustained in the long term through the maintenance of higher amounts of soil organic carbon and the production of timber products with longer life spans.

## Introduction

Reforestation efforts are promoted globally to meet the growing demand of forest products, especially in developing countries. Subtropical forests are therefore promoting the maintenance of forest cover in the region and the production of goods that improve the livelihoods of local communities. Subtropical forests cover 7–10% of the global land area, and store 40–50% of carbon present in terrestrial vegetation [1]. However, subtropical forests also account for most of current deforestation, with accumulated losses in the 1996–2010 period of ∼100 million ha [2]. Because forests are a major carbon pool, their management is crucial to develop successful policies for climate change mitigation. Carbon storage versus production of timber and non-timber forest products are seen sometimes as incompatible objectives to be implemented at the same time in a forest, but this view is being challenged [3]. Clarifying if subtropical forest plantations can support multi-objective forestry and estimating how much carbon could be stored in working forest plantations would help to maximize the outcome of forest management plans while helping to reach a more sustainable development of the subtropical regions.

New planted forests are typically established on sites that have rapid growth and access to processing facilities and growing markets. Hence, very large areas of new forest plantations have been established in subtropical countries, particularly in Latin America and parts of Asia. In spite of such rapid growth, only 3% of the world forest land is covered with productive forest plantations. However, this area expanded by 2 million ha annually in the 1990s and by 2.8 million ha in the 2000s [4].

While much attention and discussion have been devoted on controlling and reverting deforestation, how to enhance forest carbon stocks by improving forest ecosystem condition has received less emphasis until recently [5]. Reforestation has been suggested not only as an effective way to restore degraded ecosystems, but also as a way to mitigate elevated atmospheric CO_2_, hence contributing towards the reduction of climate change [3], [6]. Whether and how these efforts can maximize and sustain forest C storage largely remains unexamined [7], [8], [9]. Recent decisions by the U.N. Framework Convention on Climate Change have encouraged consideration of other ecosystem services while implementing REDD+ (Reducing greenhouse gas Emissions from Deforestation and forest Degradation) projects for forest carbon sequestration and storage. Therefore, selecting the most appropriate approaches for implementing multipurpose forest management in planted forests is just as important as growing more forests [10]. However, few incentives and policy guidelines are in place to encourage growing better forests globally, perhaps due to the lack of a clear understanding of long-term benefits from growing better forests as well as operational difficulties.

Traditional forest management practices in forest plantations, particularly in developing countries, usually involve application of short rotations, usage of monoculture and exotic species, and high-level removal of biomass, with the main purpose of maximizing short-term economic gains [11]. The ecological problems associated with those traditional forestry practices have been well documented: declines in long-term productivity [12], [13], biodiversity loss [14], increased susceptibility to insects and diseases [15], physicochemical changes in forest soils [16], and losses of other ecological services [17]. In the context of climate change mitigation, traditional forestry practices may lead to short-term carbon sequestration in wood products, but those gains are unlikely sustainable due to losses in ecosystem C linked to the above-mentioned ecological problems. Sustainable forestry practices, however, could have an important role in mitigating climate change impacts, if they are well designed and implemented [3]. For example, transformation of conifer monocultures into mixed conifer-broadleaved plantations has been considered as an efficient strategy to sustain forest productivity and restore degraded forests [18].

China has both subtropical and temperate forests, and aggressive reforestation policies have turned it into one of the five most forest-rich countries in the world, accounting for the largest gain in forested areas globally [12], [19]. However, most of ecosystems reforested during the past decades in China have been by monocultures dominated by coniferous species. It has been widely reported that timber yield declines and degradation in soil fertility in coniferous plantations over several continuous rotations (see [20], and references therein). On the other hand, in subtropical China, evergreen broadleaved forests are the main native ecosystem type in the region, with a clear potential capacity for carbon sequestration [11]. Among the native species, the broadleaf *Phoebe bournei* (Hemsley) Yang is one of the most valuable tree species in this subtropical region because of its high-quality wood properties, with significant economic and ecological benefits [21]. Sharing the same distribution area, the conifer Chinese fir (*Cunninghamia lanceolata* (Lamb.) Hook. is the most important commercial forest species in subtropical China. It has been widely planted in the southeast provinces of China [20], [22], becoming one of the most abundant planted species in the world [23].

The traditional management of Chinese fir plantations is representative of the type of management usually carried out in many other tropical and subtropical forest regions around the world. After harvesting and slash-burning the native mixed evergreen broad-leaved forests, pure plantations of fast-growing conifers were established. The plantation sites used to be abandoned after one or two rotations and allowed to regenerate naturally to mixed species stands by stump sprouting and natural seeding, which then acted as a fallow period to restore the site [24]. However, since the 1950s, the plantation area of Chinese fir has been enlarged, and this species has been repeatedly planted on the same sites without intercropping or periods of fallow. Local foresters have generally used a 25-year rotation, with variation from 20 to 30 years depending on soil nutrient abundance [25]. A trend towards rotation length shortening is being observed in the latest years, driven by increasing demand for timber products fueled by economic development and population increase in China. Continuous cultivation of Chinese fir at the same site has resulted in a well-known ecological problem: yield decline over consecutive rotations [20], [26]. With these conditionings, an important question has recently been raised in Chinese forestry with implications for the management of forest plantations in other subtropical regions of the world: can forest plantations be managed to produce forest products but in a way that also maintains or even increases ecosystem C? If so, can the differences in ecosystem C between traditional and sustainable management be quantified?

To answer these questions, in this work we tested the hypothesis that subtropical tree plantations accumulate less carbon under traditional exploitative management than under sustainable forest management, which is more similar to natural stand development. We also aim to quantify the potential increase in ecosystem C at a regional level. To test this hypothesis we first reviewed available information on C pools in subtropical forest plantations and related them to factors defining their management (species type, rotation length, stand density). Also, the literature review allowed us to identify the management prescriptions with the highest carbon storage, and to define realistic sustainable management scenarios. Finally, we estimated potential C sequestration gains through time at a regional level by comparing ecosystem C values under traditional and sustainable management regimes in subtropical plantations in China, simulating plantation growth with the forest ecosystem model FORECAST [27].

## Materials and Methods

We first carried out a literature review to test if differences in ecosystem C among subtropical plantation types exist, and also to identify the management factors that have maximized ecosystem C in those plantations in the past. Then, we used ecological modelling to estimate the average differences between traditional forest management and sustainable forest management, with consideration of the factors identified in the literature review. As ecological models need to be calibrated for specific ecosystem types [28], we used Chinese fir plantations (*Cunninghamia lanceolata* (Lamb.) Hook.) located in the subtropical region of China as a case study. Continuous repetitions of traditional practices over several rotations (monoculture, short rotations, whole-tree harvesting, and slash burning) have resulted in severe yield decline [26]. Among various possible reasons, N limitation by nutrient exports and resource competition between tree seedlings and understory are the most important factors [20]. Therefore, there is a need to improve the management of this species to establish a more sustainable and multi-purpose forestry in China.

### Literature Review of Carbon in Subtropical Plantations

We conducted a structured search of published scientific documents to define the observed range in carbon storage in the ecosystem, as well as in the belowground and aboveground fractions, and their relationships with plantation type (broadleaf or coniferous), plantation age, and stand density. We searched in the on-line databases Google Scholar (by Google Inc. http://scholar.google.com), ISI Web of Knowledge (by Thomson Reuters, http://www.isiwebofknowledge.com), China Academic Journal Database (by China National Knowledge Infrastructure, http://cnki.en.eastview.com/kns50), CAB Abstracts (by the Centre for Agricultural Bioscience International, http://www.cabdirect.org), and the databases hosted by the Canadian Forest Service Bookstore (http://bookstore.cfs.nrcan.gc.ca), the University of British Columbia’s library (http://www.ubc.ca/library), and the Public University of Navarre’s library (http://www1.unavarra.es/biblioteca).

**Figure 1:**
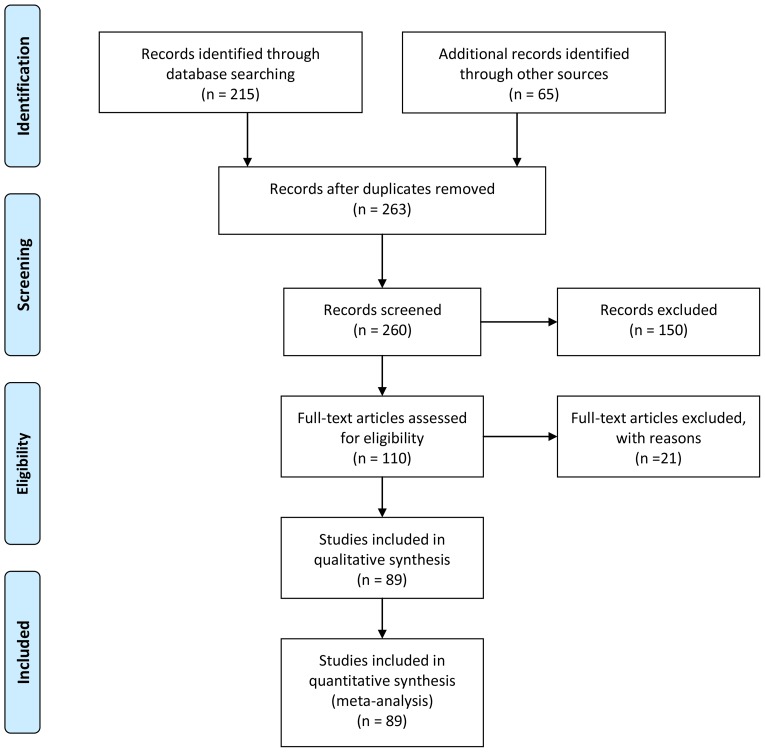
PRISMA flow diagram for the creation of the database of carbon pools in tropical and subtropical forest plantations.

**Figure 2:**
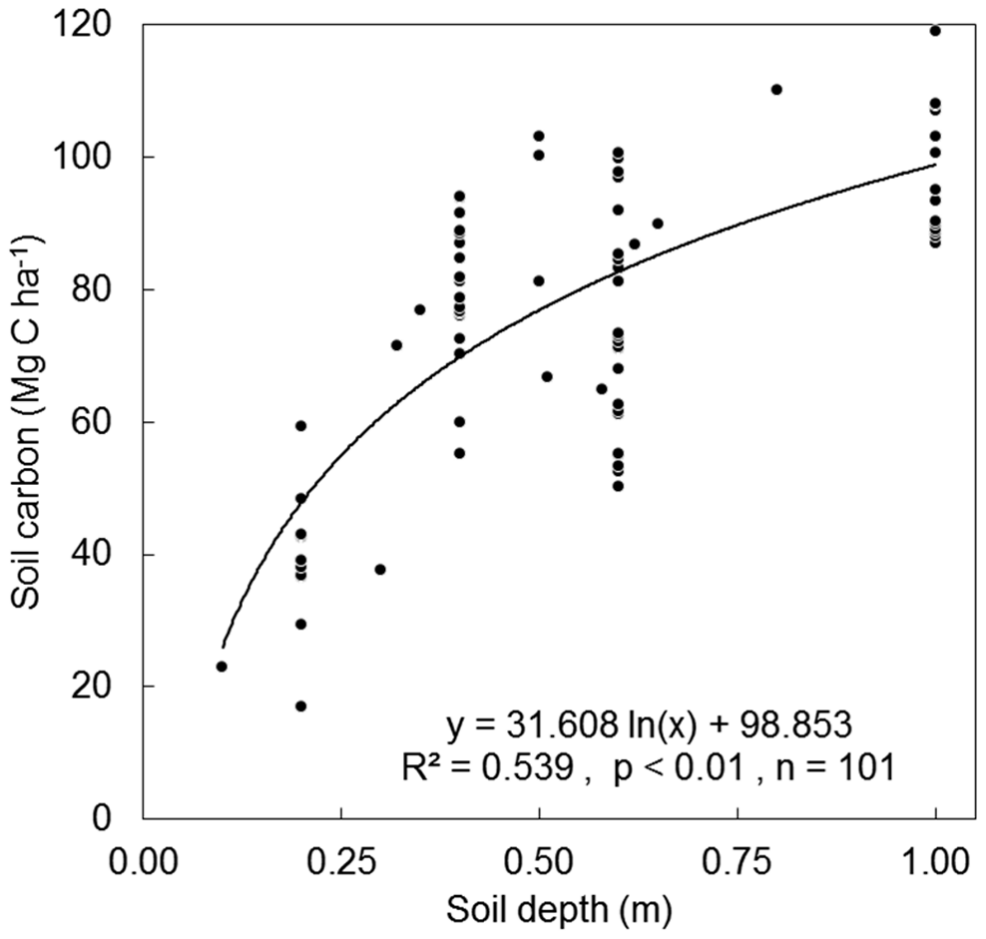
Values of soil carbon *vs.* soil depth in the reviewed literature.

We divided the population of collected documents into two groups: 1) documents reporting C pools in broadleaf or other non-coniferous forests, and 2) documents describing coniferous or mixed conifer-broadleaf plantations. The final number of documents and sites used for further analysis after a standardized selection process [29] were, for broadleaves 38 documents and 138 sites; whereas for conifers 51 documents and 160 sites (Figure 1; see the full reference list and values of carbon and management variables for each site in Material S1). For each site, we gathered data on plantation age, density, aboveground C density (MgC ha^−1^), belowground C density, and ecosystem C density. Aboveground C was calculated as tree C plus understory C (if reported). Belowground C carbon was calculated as C in tree roots (and in plant roots if reported), litter C and mineral soil C. Values from graphs were extracted using DataThief III [30]. Original values for mineral soil C were often not directly comparable, as they were measured for different soil depths. To standardize soil carbon values, we modified the method by [31] and used bulk density and soil C concentration to calculate and plot soil C content versus soil depth, obtaining a significant correlation between both variables (Figure 2). We then estimated the standardized mineral soil carbon content at 60 cm depth as the value that would correspond if soil carbon distribution in each soil followed a relationship with soil depth parallel to the regression line.

We tested the equality of means for aboveground, belowground and ecosystem C between the two types of plantations using Welch’s modified *t*-test, (a robust test that does not assume equality of variances; [32]), setting significance level at 0.05. We also carried out regressions (weighted by the error associated to each measure) of carbon density vs. plantation age and stand density, lumping together data from conifer and broadleaf plantations as initial analysis did not show improvement of the regression results by splitting the data into species groups or location (data not shown). All the statistical analyses where carried out with Sigmaplot 10.0 (Systat Software Inc.), JMP v.5.0.1 (SAS Institute, NC, USA), and S-PLUS 6.1 (Insightful Corp.).

### Simulating Ecosystem C in Planted Forests with FORECAST

**Figure 3:**
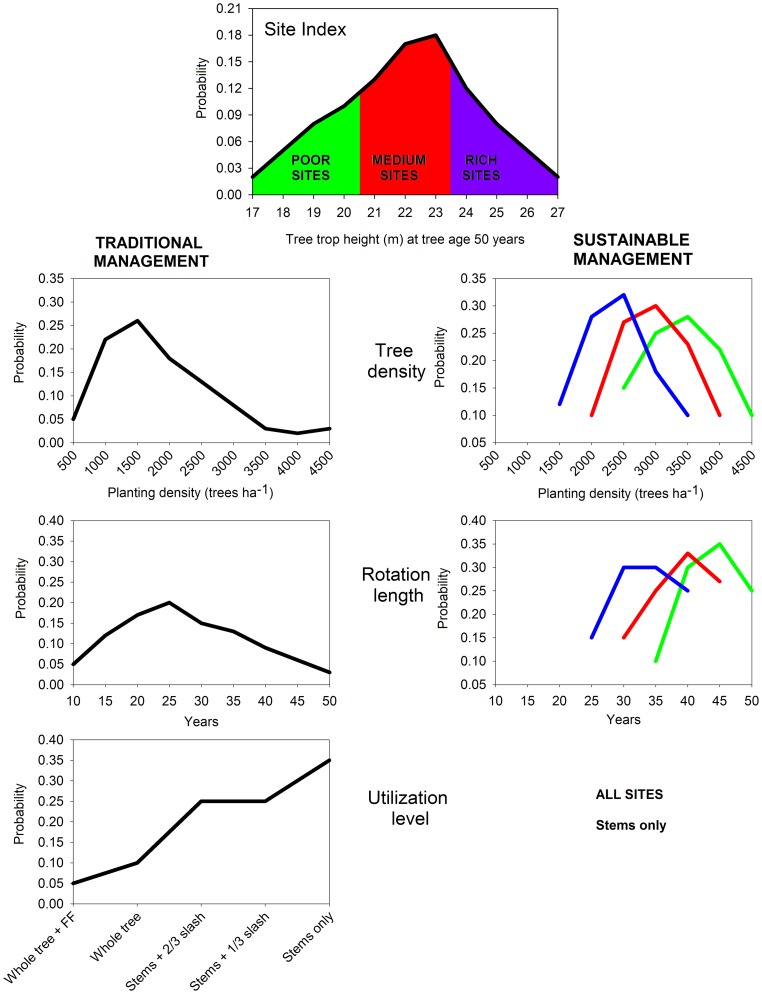
Probability distributions of the four variables defining the management scenarios. Different color lines **c**orrespond to the different site qualities as defined in the top graph. FF: Forest floor removal.

In the second part of our research, we used the results from the literature review to define probability distributions for the two main factors defining forest plantation management (stand density and rotation length) that maximized ecosystem C, and we assumed that they are indicators of more sustainable forest management. Probability distribution of site quality was defined using the distribution map of site qualities for Chinese fir in south-eastern China [33]. Probability distributions of planting density, rotation length and utilization level for traditional management were also created for both sets of simulations after consultations with panels of local experts composed by academics, forest officers, and forest managers (Figure 3). The panel of local experts indicated the need for creating different management scenarios based on site quality for sustainable management. Hence, probability distributions of tree density and rotation length were different in each site quality for sustainable management, but they were the same in all sites under traditional management.

Using the probability functions, we constructed 200 Monte Carlo projections for each set of simulations (traditional management and sustainable forest management). Each run simulated a management scenario (a random combination of site quality, rotation length, and stand density). In traditional management, all trees planted were Chinese fir. In sustainable management, probability distributions show total tree density, which was composed by Chinese fir and *Phoebe bournei* in a 1∶1 ratio, the most suitable species mix for carbon sequestration [21].

We then simulated each scenario for 120 years with the ecosystem model FORECAST, which is a stand-level model specially designed for assessing the effects of various forest management practices on forest carbon storage and sequestration. FORECAST has been successfully tested and applied in many world forests before, including tropical and subtropical forests [28].

FORECAST is a management-oriented, deterministic, non-spatial stand-level forest growth and ecosystem dynamics simulator that operates at annual time steps. The model has been described in detail before [27], [28], and therefore only a summary of the main driving function used to calculate tree growth is provided in Material S1. The model uses a mass balance approach to estimate ecosystem nutrient circulation, and how nutrient availability limits tree growth together with available light in the canopy. FORECAST has three application stages: 1) assembling calibration data and generating historical rates of key ecosystem processes; 2) model initialization by establishing the ecosystem condition for the beginning of a simulation run; and 3) simulation of tree and plant growth.

Input data came from sites covering the observed range of Chinese-fir plantation growth, qualitatively described as poor, medium and good (Table 1). The sites were scaled quantitatively as 17, 21 and 27 (from poor to good, based on canopy top height in m at age 50 years) to represent a relative index of tree growth required for extrapolation within the model. Tree data on biomass, mortality, stand density, tree height, canopy height, nutrient concentrations of live tissues and other data were based on reported values for similar Chinese-fir plantations [25], [34], [35], [36], [37], [38], [39], [40], [41], [42], [43].

**Table 1:** Values used to calibrate FORECAST parameters related to Chinese-fir.

Parameter	Unit	Rich site	Poor site
**Chinese fir parameters**			
Nitrogen concentration in needles young/old/dead	%	1.53/1.36/1.13	1.21/1.11/0.93
Nitrogen concentration in stem sapwood/heartwood	%	0.14/0.03	0.12/0.03
Nitrogen concentration in bark live/dead	%	0.44/0.27	0.37/0.24
Nitrogen concentration in branches live/dead	%	0.67/0.52	0.55/0.47
Nitrogen concentration in root sapwood/heartwood	%	0.37/0.06	0.35/0.06
Nitrogen concentration in fine roots live/dead	%	1.17/0.97	0.96/0.79
Shading by maximum foliage biomass	% of full light	8	30
Soil volume occupied at maximum fine root biomass	%	100	95
Efficiency of N root capture	%	98	100
Retention time for young/old foliage/dead branches	years	1/2/40	1/2/40
Fine roots turnover	year^−1^	0.95	1.35
**Decomposition rates**			
Sapwood (by litter age)	% year^−1^	1–5 years (2.0); 6–10 years (10.0); 11–15 years (30.0); 16–20 years (20.0); >20 years (4.0)	
Heartwood	% year^−1^	1–10 years (0.4); 11–15 years (10.0); 16–25 years (15.0); 25–40 years (10.0); >40 years (2.0)	
Bark	% year^−1^	1–5 years (2.0); 6–20 years (12.0); 20–40 years (20.0); >40 years (4.0)	
Branches and large roots	% year^−1^	1–5 years (10.0); 6–10 years (45.0); 11–15 years (35.0); >15 years (4.0)	
Needles (poor site)	% year^−1^	1–2 years (20.0); 3–5 years (30.0); 6–10 years (40.0); >10 years (2.0)	
Needles (good site)	% year^−1^	1–2 years (27.0); 3–5 years (30.0); 6–10 years (40.0) >10 years (3.0)	
Fine roots	% year^−1^	1–2 years (30.0); 3–4 years (50.0); >4 years (9.0)	

Decomposition rates indicate the mass loss in one year as a fraction of the initial mass at that year. See text for list of bibliographical sources for model calibration.

Model calibration data for *Phoebe bournei* forests (Table 2) came from literary sources and field data [21]. They included data of biomass accumulation and stand density [44], [45], [46], [47], [48], [49], [50], decomposition rates [51], [52], [53], [54], soil nutrients under different site conditions [55], [56], [57], and photosynthetic efficiency [58], [59], [60], [61]. Understory was simulated as grass and shrub complexes [20]. Published data were used to characterize shrub biomass, height, tissue nutrient concentrations and other relevant data [62], [63], [64], [65] (Table 3).

**Table 2:** Values used to calibrate FORECAST parameters related to *Phoebe bournei.*

Parameter	Unit	Rich site	Poor site
**Chinese fir parameters**			
Nitrogen concentration in leaves young/old/dead	%	1.35/0.92/0.69	1.15/0.80/0.67
Nitrogen concentration in stem sapwood/heartwood	%	0.18/0.04	0.16/0.04
Nitrogen concentration in bark live/dead	%	0.55/0.47	0.49/0.33
Nitrogen concentration in branches live/dead	%	0.33/0.29	0.28/0.26
Nitrogen concentration in root sapwood/heartwood	%	0.37/0.29	0.34/0.27
Nitrogen concentration in fine roots live/dead	%	0.81/0.57	0.65/0.56
Shading by maximum foliage biomass	% of full light	8	25
Soil volume occupied at maximum fine root biomass	%	100	100
Efficiency of N root capture	%	100	100
Retention time for young/old foliage/dead branches	years	1/0.5/30	1/0.5/30
Fine roots turnover	year^−1^	1.00	1.15
**Decomposition rates**			
Sapwood (by litter age)	% year^−1^	1–3 years (0.1); 4–15 years (2.0); 15–20 years (12.0); >20 years (9.0)	
Heartwood	% year^−1^	1–3 years (0.1); 4–15 years (2.0); 15–20 years (12.0); 20–40 years (9.0); >40 years (2.0)	
Bark	% year^−1^	1–10 years (20.0); 11–20 years (40.0); 21–30 years (35.0); >30 years (2.0)	
Branches and large roots	% year^−1^	1–5 years (15.0); 6–15 years (35.0); 16–20 years (45.0); >20 years (15.0)	
Leaves (poor site)	% year^−1^	1–2 years (45.0); 3–5 years (35.0); >6 years (20.0)	
Leaves (good site)	% year^−1^	1–2 years (50.0); 3–5 years (40.0); >6 years (20.0)	
Fine roots	% year^−1^	1–5 years (45.0); >6 years (35.0)	

Decomposition rates indicate the mass loss in one year as a fraction of the initial mass at that year. See text for list of bibliographical sources for model calibration.

**Table 3:** Values used to calibrate FORECAST parameters related to the shrub complex and soil processes.

Shrub complex parameters	Unit	Rich site	Poor site
Nitrogen concentration in leaves live/dead	%	1.68/1.38	1.12/0.76
Nitrogen concentration in stems live/dead	%	0.48/0.14	0.32/0.10
Nitrogen concentration in rhizomes live/dead	%	1.14/1.05	0.76/0.70
Nitrogen concentration in roots live/dead	%	1.10/0.84	0.73/0.56
Shading by maximum foliage biomass	% of full light	0.20	0.45
Soil volume occupied at maximum fine root biomass	%	60	50
Efficiency of N root capture	%	99	99
Transfer from live to dead stem/rhizomes/roots	% year^−1^	20/20/30	20/20/40
Retention time for foliage	years	1	1
**Decomposition rates**		Litter age in years (decomposition rate in %)	
Foliage (poor site)	% year^−1^	1 year (80.0); 2–3 years (60.0); 4–5 years (50.0); >5 years (2.0)	
Foliage (rich site)	% year^−1^	1 year (95.0); 2–3 years (60.0); 4–5 years (50.0); >5 years (2.0)	
Stems & roots	% year^−1^	1 year (20.0); 2–3 years (30.0); 4–5 years (40.0); >5 years (2.0)	
**Soil parameters**		**Rich site**	**Poor site**
Nitrogen concentration in slow/fast humus	%	3.00/1.40	3.0/1.40
Decomposition rate slow/fast humus	% year^−1^	0.15/2.00	0.15/2.00
CEC soil (CEC humus)/AEC	kg N ha^−1^	80.0 (0.2)/20.0	40.0 (0.2)/5.0
Atmospheric deposition/non-symbiotic fixation	kg N ha^−1^ year^−1^	4.9/1.0	4.9/1.0
Non-symbiotic N fixation rate	Kg N ha^−1^	2.5	3.0

Decomposition rates indicate the mass loss in one year as a fraction of the initial mass at that year.

Data describing soil processes and nutrient inputs in precipitation and slope seepage, mineral soil cation and anion exchange capacities, humus mass, nutrient concentrations in litterfall, litter decomposition rates and others. were derived from literature [38], [39], [40], [66], [67], [68], [69], [70], [71] (Table 3). To establish initial site conditions the model was run in set-up mode, forcing the model to match the observed site conditions [72], [73]. Initial conditions were created for each site quality, by running several cycles of tree growth ending with stand-replacing windthrow, as typhoons are the most common natural disturbance in this region [74]. A library of initial conditions was created with initial soil organic matter values (humus+litter) ranging from 90.9 to 296.3 Mg ha^−1^ for the poorest and richest sites, respectively.

Published field data on several chronosequences at different site qualities were used to evaluate FORECAST performance for Chinese fir and *Phoebe bournei* plantations (see Material S1). After obtaining a satisfactory model performance, we run the model for the 400 randomly-generated scenarios described above. A sub-set of documents gathered during the literature review dealing with Chinese fir plantations was also used to evaluate the model performance for ecosystem C.

## Results

**Figure 4:**
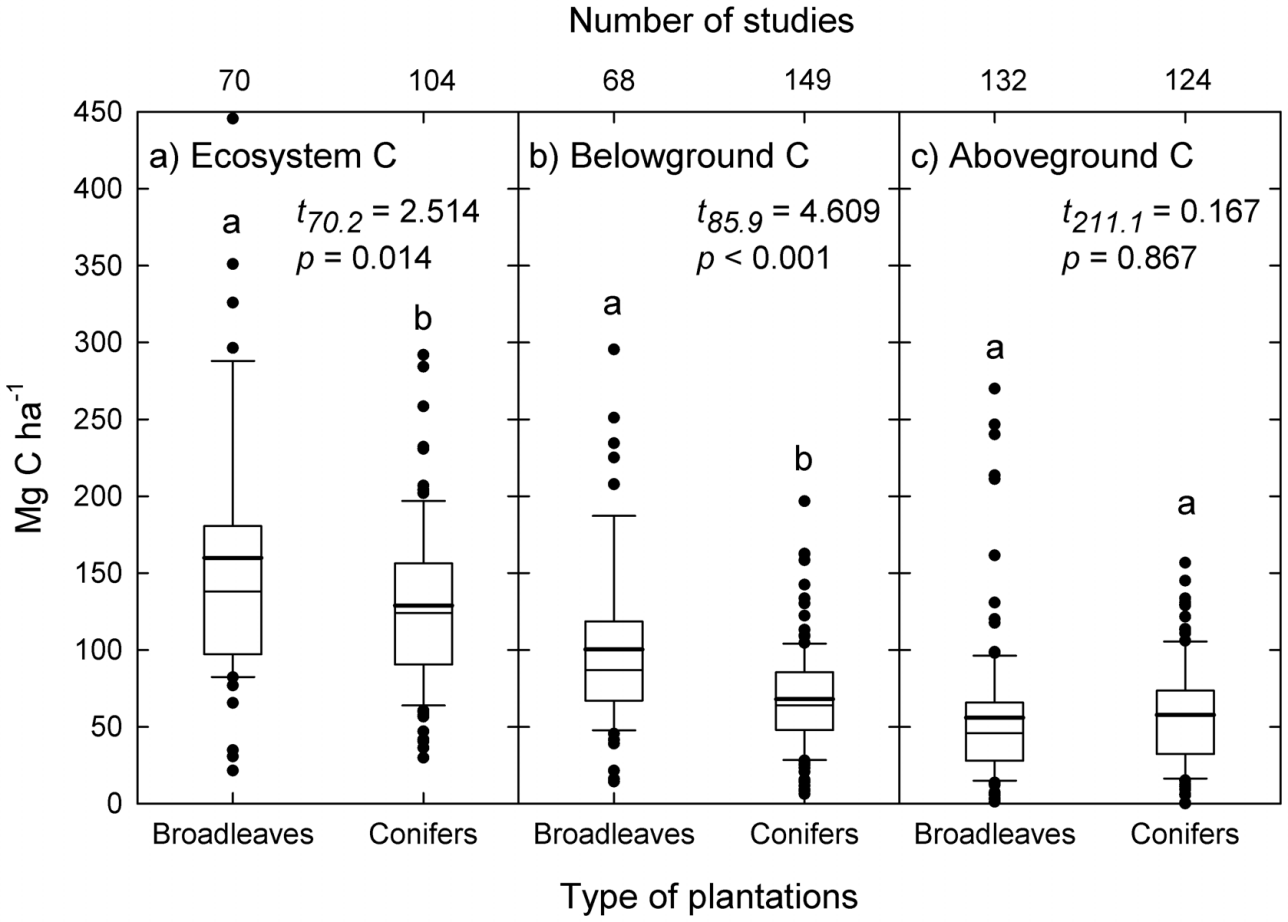
Distribution of C storage in published bibliography for different subtropical plantation types. The thick horizontal line indicates the average of all observations. Letters indicate significant differences with Welch’s *t* at *P*<0.05. See Material S1 for the complete list of references used.

**Figure 5:**
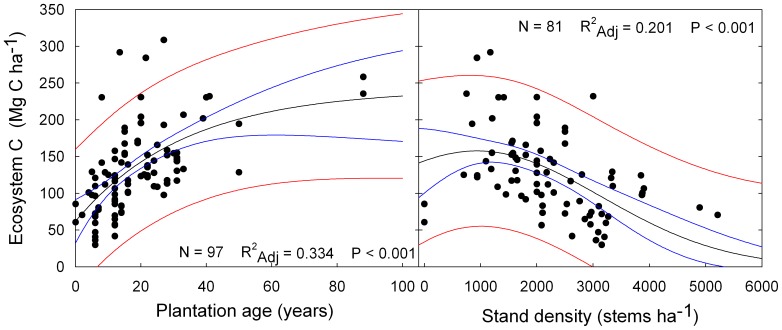
Carbon stocks in subtropical plantations for different A) rotation ages, and B) stand densities. Black lines indicate the best-fit model, blue lines 95% confidence interval and red lines 95% prediction intervals.

From our review, 23.9% of the studies on bradleaves used local native species, but *Populus* spp. (19.6%), *Tectionia sp.* (13.1%) and *Eucalyptus* spp. (8.10%) were the most common commercial species used. For conifers, 81.4% of the studies reported data from *Cunninghamia* sp. plantations, and 14.3% came from *Pinus* spp. plantations. Our results showed that subtropical broadleaves plantations can, on average, sustain higher carbon densities than subtropical conifer plantations (Figure 4). The differences, however, are mostly caused by differences in belowground C, whereas differences among plantations types in aboveground C are non-significant. Not surprisingly, the trend in subtropical forest plantations is to increase ecosystem carbon storage as plantation age increases. This is basically the result of increase in tree size, and accumulation of litter and coarse woody debris biomass. This trend, however, seems to slow down over time, with little expected gains beyond plantation age ∼90, as described by the significant exponential model (Figure 5). Ecosystem C initially increases as plantation density increases, reaching a peak at about 1500–2500 stems ha^−1^. For higher densities the trend is to reduce ecosystem C (Figure 5).

During the simulation runs, the most frequent scenario for sustainable forest management was 3000 trees ha^−1^ (1500 Chinese fir, 1500 *P. bournei*), rotation length of 40 years and stem-only harvesting. For traditional management, the most frequent scenario was 1500 trees ha^−1^ (Chinese fir only), rotation length of 25 years, stem plus slash removal, and site preparation by understory burning. Comparing these two scenarios when run for 240 years, we can see how plantations managed for sustainable, long-term goals, maintain ecosystem productivity higher and during longer periods that traditional intense management.

**Figure 6:**
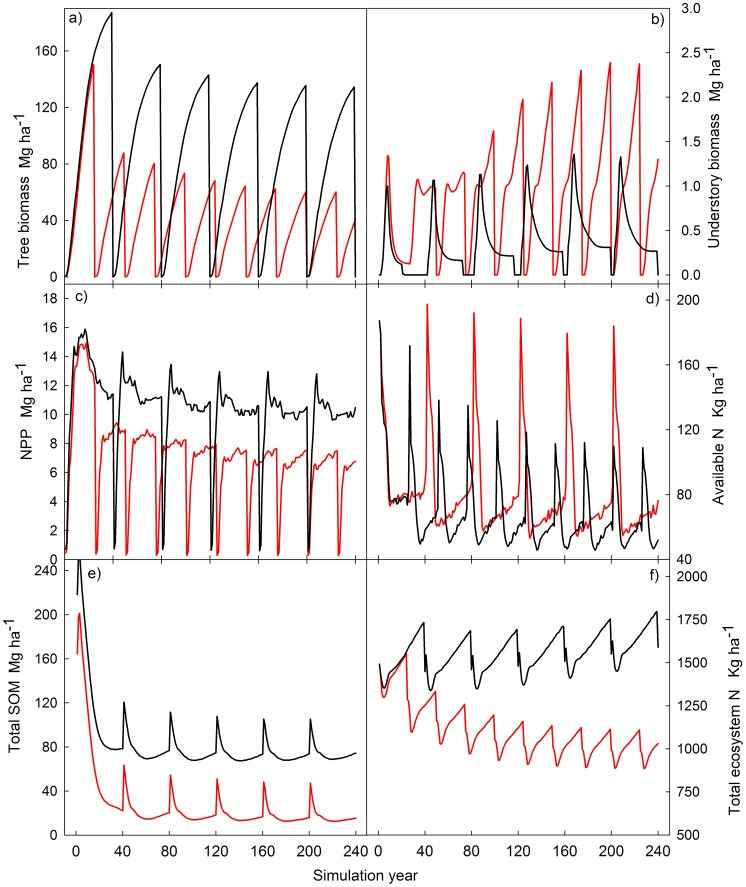
Change in ecological variables in Chinese fir plantations in 240 years of simulation under the average plan two types of management. Current (red line) 25-year rotation, whole-tree harvesting, 1500 trees ha^−1^ Chinese fir planted; and close-to-nature management (black line) 40-year rotations, stem-only harvesting, 1500 trees ha^−1^ Chinese fir +1500 trees ha^−1^ *Phoebe bournei.*

Yearly net primary productivity under traditional management quickly drops in each consecutive rotation, and as a consequence tree biomass (and the associated merchantable volume) also decreases rapidly over time (Figure 6). In addition, there is an increasingly stronger competition with understory. Although with traditional management higher peaks of available soil N are achieved than in the sustainable management, the total amount of ecosystem N decreases over time. On the other hand, under sustainable management higher levels of soil organic matter are sustained (Figure 6).

**Figure 7:**
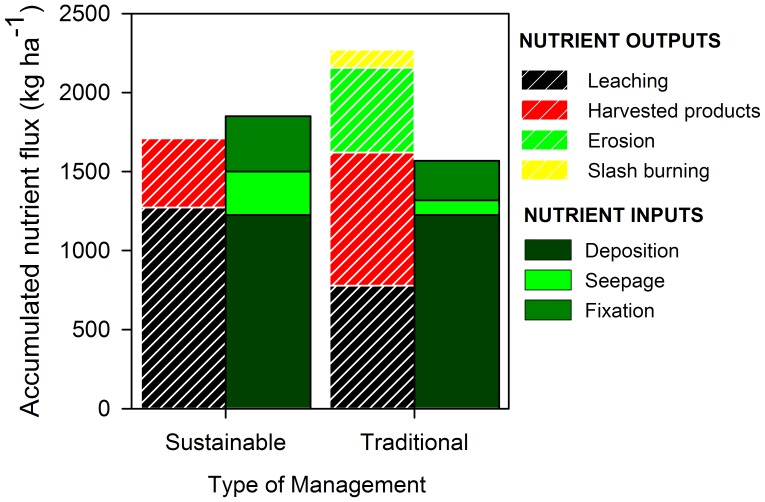
Accumulated N inputs (solid bars) and outputs (dashed bars) in Chinese subtropical plantations after 240 years of management under two most typical management regimes for traditional and sustainable plantations.

When all the inputs and outputs are accumulated through the 240 years of simulation, it is clear how current management is depleting ecosystem N reserves. On the other hand, sustainable management can maintain and even increase N pools (Figure 7). Traditional management removes N from the ecosystems during the site preparation (directly through slash-burning and indirectly by erosion caused by heavy machinery and losses of plant cover), with the export of harvested products, and by N leaching. Under sustainable forest management, slash-burning is not used and erosion is greatly reduced as practices affecting soil stability and plant cover are minimized. N removal in harvested products is also lower as only stems (with low N concentrations) leave the plantations. On the other hand, as N reserves under sustainable management are higher, leaching losses are also higher, just because the soil N pool is bigger than in traditional management. All flows considered, N outputs with current practices are not only higher, but N inputs are also smaller, as there is less N fixation due to the reduced biological activity in sites of lower productivity. Also, because any given stand gets less N inputs by seepage as it is surrounded by stands that also have lower N capitals.

**Figure 8:**
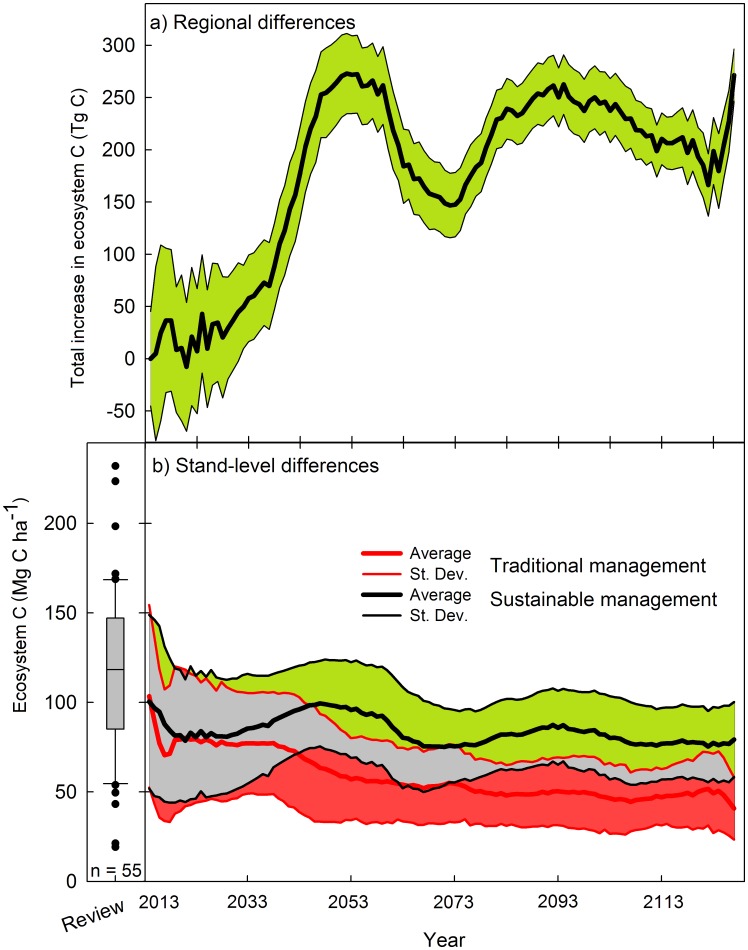
Total carbon gains when switching from current traditional intensive forestry to sustainable forest management at an annual rate of 10% of total area dedicated to Chinese-fir plantations (panel A). Ecosystem carbon density in Chinese fir plantations under current intensive management (red lines), and sustainable management (green lines) (panel B). Thick lines represent average and color areas represent one standard deviation. The grey area indicates values of ecosystem C that can be achieved by either sustainable or traditional management plans. The box-plot in the bottom-left summarizes the values obtained from a review of 55 published peer-reviewed papers on carbon stocks in Chinese fir plantations (see Material S1 for the complete reference list).

When simulating how the C pools in Chinese fir stands would evolve over the 21st century, our results showed that if sustainable forestry practices were introduced at a rate of 10% of the total planted area, the region would keep on average a higher and sustained level of carbon storage over the long term, compared to traditional management (Figure 8). At the beginning of our scenarios a large overlapping range for ecosystem C between traditional and sustainable plantations can be seen (grey area). Such fact indicates that some time is needed for the sustainable management to significatively reduce C loses compared to traditional management. This is not surprising as productivity losses are accumulative and occur mostly at the change of rotation. However, by the middle and until the end of the simulated 120 years the overlap has been greatly reduced. By that time, only the sites with the highest C contents and managed traditionally would be comparable to the poor sites under sustainable management. Such increase in carbon density could be 12% by 2020. This difference would grow over time, reaching 17%, 52%, 79%, and 67% greater carbon density under sustainable forest management after 25, 50, 75 and 100 years, respectively. The average weighted carbon density increase over the 100-year simulation period was 67.5%.

As a way to validate our estimations at the stand level, when analyzing the average and 95% confidence intervals for the whole set of projected ecosystem C values they were always inside the range of reported values for Chinese-fir plantations (Figure 8), providing confidence on our estimations.

Stand-level differences between management regimes would have large regional implications. Extrapolated to the whole area dedicated to Chinese fir plantations (7,046,520 ha [11]), together with the plan of substituting traditional by sustainable management implemented at a ratio of 10% of the land every year, the increase in ecosystem C stored in the mixed Chinese fir plantations could reach 261.2 TgC by 2050 (Figure 8). After that year, the differences in ecosystem carbon storage between management options show a temporal decline. This is as a consequence of the harvesting of sustainable forest plantations primarily clustered around the years 40 to 60. The second rotation growth would again produce an increase in stored C, which would remain above 200 TgC after 120 years of simulation.

## Discussion

Subtropical plantations have been described as the most effective for C sequestration [75], [76], [77]. In subtropical regions, local broadleaved species are usually planted, but the available data on C stocks in subtropical plantations came mostly from the five genuses more widely planted around the globe: *Eucalyptus, Populus, Tectonia, Pinus* and *Cunninghamia*. These are the most valuable among the fast-growing species currently used, and our results show that selecting among the species type (broadleaves or conifers) can produce significant differences in C pools depending on plantation type and management regime. Ecosystem C increases with tree age in subtropical plantations, as these systems develop ecological structures that make them closer to natural forests over time [78]. Older plantation age has also been associated to higher soil carbon levels [79]. Therefore, increasing rotation length should be encouraged when implementing sustainable forestry plans for increasing carbon stocks, as well as generating more biophysical structure for higher niche diversity and its associate biodiversity.

[80] have pointed out that density plays an important role in the productivity of mixed stands, with higher productivities in mixed stands than in monocultures at medium to high densities (>1500 trees ha^−1^). Our results also indicate that density should be maintained at medium values, avoiding excessive inter-tree competition for resources such as light, water and nutrients that could reduce stand productivity, but also avoiding low densities that may leave some resources unused by trees. Our estimates also fit with several field experiments in mixed plantations of Chinese fir with other trees species, which have consistently shown higher biomass and C density in mixed stands with a tree density ratio of 1∶1 for Chinese fir/companion species, for total densities in the 2000–3000 trees ha^−1^ range [81], [82], [83], [84].

Our results indicate that managing plantations designed for multiple values could sustain higher carbon storage potentials if they include broadleaves, are managed for rotations lengths of 25–50 years, and have planting densities of 1500–2500 stems ha^−1^. Our estimations corroborate previous field research that have reported maximum biomass values in mixed Chinese fir plantations for similar rotation and density ranges, reporting little gain or even biomass decrease with plantation densities above 3000 trees ha^−1^ [82], [85]. In this regard, forest plantations seem to have important functional similarities with native forests, where intermediate tree densities have been reported as the ones reaching the carrying capacity of the ecosystem, without surpassing it [86].

Differences in C density among management types can be better understood by comparing the typical (most frequent in our set of simulations) management regimes for traditional and sustainable forest management practices. Depletion in ecosystem N reserves reduces ecosystem productivity, which in turn produces a long-term decrease in tree biomass [87]. This productivity reduction is much more pronounced under traditional practices, as shorter rotation lengths accumulate higher and faster nutrient exports. In addition, short rotations and lower stand densities produce an under-canopy environment with more light available and therefore more suitable for understory growth (Figure 6). In these plantations, understory grass and shrubs usually sprout from rhizomes and other underground structures, rather than from seeds [20]. This phenomenon provides a competitive advantage against planted tree seedlings, which is exacerbated by the advantage of understory’s lower nutrient needs in increasingly nutrient-poor sites as a consequence of intensive organic matter extraction after harvesting [88], slash burning [89], and N leaching [90] (Figure 7).

Reductions in soil organic matter and its associated sources of nutrients, water retention and physical properties would also be more severe under traditional management [16], leading to the already reported yield decline in Chinese fir plantations [26]. On the other hand, our results show that using longer rotations, moderately higher stand densities, and mixing conifers and broadleaves would prevent site productivity decline. With more sustainable management, the ecosystem would maintain and even can increase N reserves by keeping a positive N balance, an indicator of ecological sustainability of forest management in the long-term [26], [91].

Theoretical forest ecology indicates that in tree species mixtures, if each species has different growing patterns (differential shade tolerance, physical separation of canopies, phenological differences, successional separation, and differences in soil resource utilization), inter-specific competition may be reduced. Growing evidence indicates a generalized pattern in mixed forests, in which niche separation at both canopy and root levels leads to a more complete use of site resources [80], [92], [93], [94]. This phenomenon has already been reported in mixed tropical plantations [95], [96], [97]. Therefore, resources in the mixed stand are used more efficiently, hence total stand productivity increases. This phenomenon is due to two facts: complementary resource use between species and facilitative improvement in nutrition in mixtures with a nitrogen-fixing species [93], [95], [98].

Our results fit with this theoretical background, showing that in Chinese fir plantations, implementing mixtures with *Phoebe bournei* will not only increase productivity, but it will also prevent SOM and N losses and their associated yield decline over time. The additional increase in sequestered ecosystem C is another positive effect of species mixtures in these sites. Similar results have also been reported when simulating boreal [99], temperate [73] and tropical [100] forests.

Forest management can greatly affect net C exchange with the atmosphere, not only because it changes ecosystem C pools, but also because the C removed from the ecosystem during harvesting is stored in wood products, such as paper, cardboard, furniture, structural timber, etc. [101]. We have shown that subtropical plantations managed under sustainable forest management can store more ecosystem carbon. However, their net effect on C sequestration depends on forest types, their management regimes, the type of wood products produced, and the efficiency of biomass conversion, as well as assumptions about how the wood and wood residues will substitute for other products with greater GHG emissions and for fossil energy [102].

In Chinese fir plantations under traditional management, most of the production goes to the market as small diameter trunks. Over time, harvested sizes become increasingly smaller as plantation sites lose productivity due to the nutrient losses and competition with understory. However, using a more sustainable forest management, harvesting rates are reduced as it takes more time to complete the harvesting turn, but on the other hand harvested tree sizes are bigger and stay bigger through several rotations. These wood products are expected to reach better prices and last longer because they will be used more as structural timber and high quality furniture [103]. The increased ecosystem C storage, combined with the longer lifespan of higher quality wood products, could compensate the reduced harvesting rates compared to traditional management. Although a life-cycle analysis is needed to test this hypothesis, previous work has shown that secondary forests allowed growing rather than being harvested and their wood products stored could result in lower net CO_2_ emissions [104].

Effects of forest management actions on carbon should be examined for large areas and over long time periods [105]. In the case of China, government’s commitment to increase forest cover by 40 million ha with new plantations may not have an impact on carbon storage as important as intended unless better forest management is also implemented [106]. In this context, applying a more ecologically-based forestry could make a significant difference. Assuming those new 40 million ha were evenly distributed among all Chinese forest types, there could be 17.55 million ha of new subtropical plantations by 2020 [8]. If the differences between sustainable and traditional forestry were similar to the ones we have estimated for Chinese fir plantations, a more ecologically-based forestry implemented in Chinese subtropical plantations could add 650.8 TgC by 2050.

Previous research on long-term C storage under sustainable forest management regimes in Chinese temperate forests have estimated ecosystem C densities of 600–750 MgC ha^−1^ in poplar (Dai *et al*. under review), 450–800 MgC ha^−1^ in spruce [107], and 200–325 MgC ha^−1^ larch plantations [108]. In all cases, estimates C densities for temperate plantations were higher than our estimates for subtropical plantations. Therefore, it seems reasonable to assume that differences between traditional and sustainable forest management regimes in temperate plantations are at least similar to the ones calculated for subtropical plantations. Under this assumption, C storage in Chinese plantations could potentially be increased by 1,482.8 TgC by 2050. These estimations could even be conservative, as temperate plantations have on average higher levels of ecosystem carbon stocks [109].

In terms of emissions, applying sustainable forestry to all new Chinese plantations could potentially lead to an average net increase in stored C of 0.04 PgC y^−1^ in 2050, equivalent to offsetting 1.91% of China’s annual carbon emissions in 2010 [110]. More importantly, this carbon increase would be sustainable with opportunities to maintain other ecological services and functions linked to increased biodiversity and less degraded forest soil [3].

In conclusion, our results show that applying a more ecologically-based forestry would have positive impacts not only on long-term plantations productivity, but it can also be a better strategy to implement climate change mitigation programs. Carbon stored in plantations could be further increased if sustainable forestry practices were combined with other practices not examined here, such as genetic improvement of trees, landscape design, etc., although the ecological sustainability of such practices should also be assessed. Finally, socio-economic incentives for farmers to extend rotations and to market non-conifer timber and other products would also be important.

## Supporting Information

Material S1 Additional information describing the FORECAST model, its validation for subtropical forests in China, and the list of references used for the review on carbon pools in subtropical planted forests.
(PDF)
