# Supplementary material for: Significant Increase in Ecosystem C Can Be Achieved with Sustainable Forest Management in Subtropical Plantation Forests
Source: PLoS One. 2014 Feb 24;9(2):e89688. doi: 10.1371/journal.pone.0089688 (PMC3933645; doi:10.1371/journal.pone.0089688)
Supplement: Material S1 — Additional information describing the FORECAST model, its validation for subtropical forests in China, and the list of references used for the review on carbon pools in subtropical planted forests. (PDF) [file pone.0089688.s001.pdf]

SUPPORTING INFORMATION FOR THE ARTICLE

Significant increase in ecosystem C can be achieved with sustainable forest management in subtropical plantation forests

Material S1

Xiaohua Wei [1], Juan A. Blanco [2, *]

[1] Department of Earth and Environmental Sciences, University of British Columbia (Okanagan), Kelowna, British Columbia, Canada

[2] Departmento de Ciencias del Medio Natural, Universidad Pública de Navarra, Pamplona, Navarra, Spain

* Corresponding author: Juan A. Blanco

Tel: +34 948 16 98 59

Fax: +34 948 16 89 30

E-mail: juan.blanco@unavarra.es

1. FORECAST model description

The ecosystem management simulation model FORECAST (Kimmins and others 1999) has been used as a long-term management evaluation tool in several types of forest ecosystem (e.g., Morris and others 1997; Wei and others 2000, 2003; Seely and others 2002; Welham and others 2002), including tropical and sub-tropical plantations (Bi and others 2007; Blanco and González 2010). Evaluation exercises have demonstrated the reliability of this model (Blanco and others 2007; Seely and others 2008; Blanco and González 2010). FORECAST was specifically designed to examine the impacts of different management strategies or natural disturbance regimes on long-term site productivity. The projection of stand growth and ecosystem dynamics is based on a representation of the rates of key ecological processes regulating the availability of, and competition for, light and nutrient resources (Figure S1). The rates of these processes are calculated from a combination of historical bioassay data (biomass accumulation in component pools, stand density, etc.) and measures of certain ecosystem variables (e.g. decomposition rates, photosynthetic saturation curves) by relating 'biologically active' biomass components (foliage and small roots) to calculations of nutrient uptake, the capture of light energy, and net primary production. Using this 'internal calibration' or hybrid approach, the model generates a suite of growth properties for each tree and plant species to be represented. These growth properties are subsequently used to model growth as a function of resource availability and competition (Kimmins and others 1999). They include (but are not limited to): 1) Photosynthetic efficiency per unit foliage biomass based on relationships between foliage biomass, simulated self-shading, and net primary productivity after accounting for litterfall and mortality; 2) Nutrient uptake requirements based on rates of biomass accumulation and literature- or field-based measures of nutrient concentrations in different biomass components on different site qualities; 3) Light-related measures of tree and branch mortality derived from stand density input data in combination with simulated light profiles. Light levels at which foliage and tree mortality occur are estimated for each species.

Soil fertility in FORECAST is represented based on empirical input data describing decomposition (mass loss) rates and changes in chemistry as decomposition proceeds. These data allow for the calculation of nutrient release from litter and humus (Figure S1). Nutrient uptake demands of different species on sites of different fertility are based on observed biomass accumulation rates and tissue nutrient concentrations on these sites, allowing for internal cycling of nutrients. The calculated uptake demand by the observed growth rates on sites of different productivity permits a

definition of nutritional site quality. This assumes that moisture is not the major limiting factor, or that, if it is limiting, it acts dominantly through soil processes that determine nutrient availability. In the humid climates that characterise the Chinese fir region this assumption is felt to be reasonable.

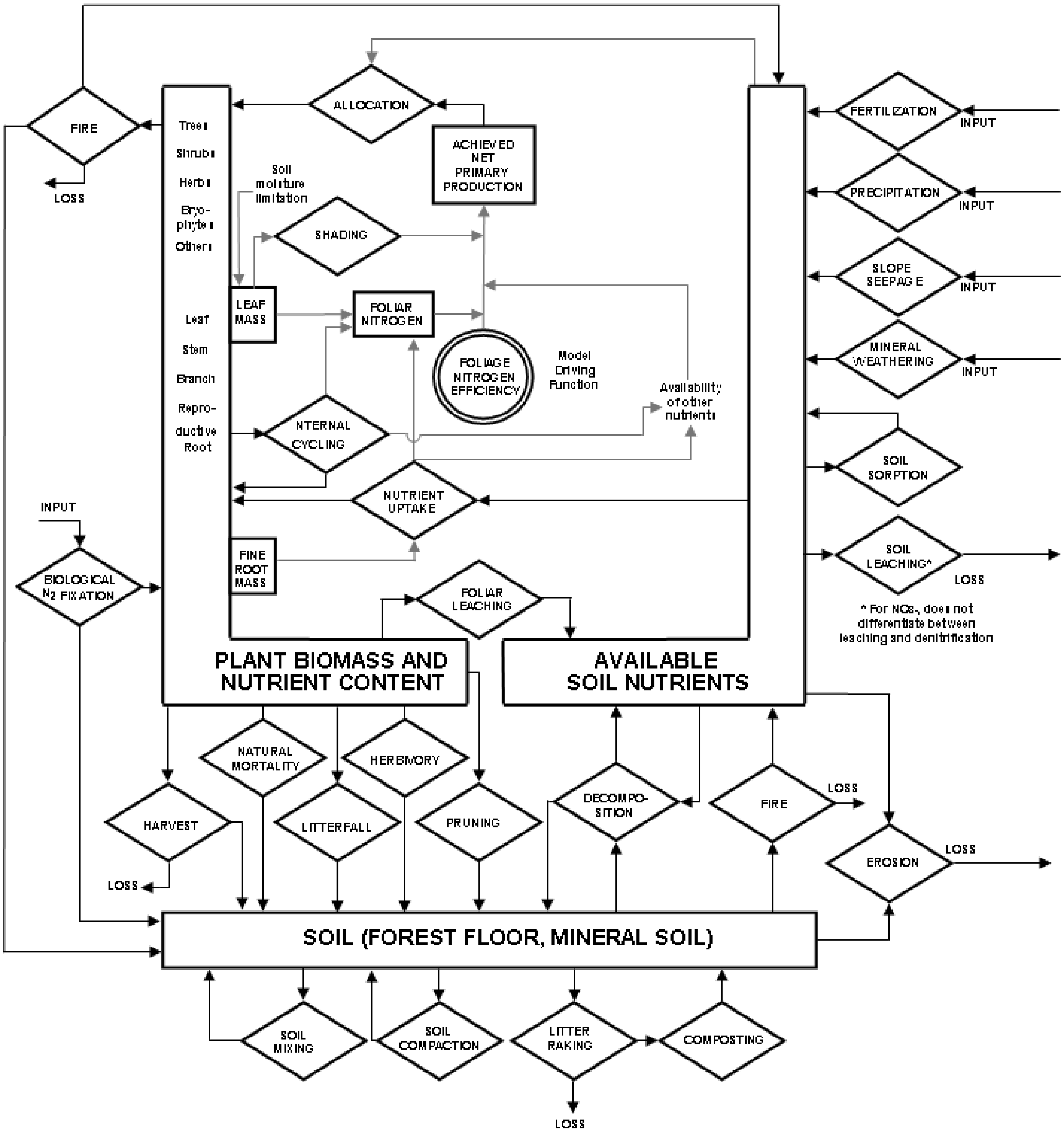

Figure S1. A schematic representation of the ecosystem compartments and transfer pathways represented in FORECAST (adapted from Kimmins et al. 1999).

Carbon allocation in response to soil fertility and tree/plant nutrition is based on empirical biomass ratios and biomass turnover rates (e.g., number of years of leaf retention for evergreens) for sites of different fertility (e.g., different site nutritional quality), and on literature or locally-obtained values for variation in fine root turnover along fertility gradients. FORECAST performs many of its calculations at the stand level but includes a submodel that disaggregates stand-level productivity into the growth of individual stems with user-inputted information on stem size distributions at different stand ages. Top height and diameter at breast height (DBH) are calculated for each stem and used in a taper function to calculate total and individual gross and merchantable volumes.

2. Model application

FORECAST has four stages in its use: 1) data assembly, input and validation; 2) establishing the ecosystem condition for the beginning of a simulation run (by simulating the known or assumed history of the site); 3) defining a management and/or natural disturbance regime; 4) simulating this regime and analyzing model output. The first two stages represent model calibration. Calibration data are assembled that describe the accumulation of biomass (above and below-ground components) in trees and minor vegetation for chronosequences of stands developed on sites that vary in nutritional quality. Tree biomass and stand self-thinning rate data are often generated from the height, DBH and stand density output of traditional empirical growth and yield models in conjunction with species-specific component biomass allometric equations. To calibrate the nutritional aspects of the model, data describing the concentration of nutrients in the various biomass components are required. FORECAST also requires data on the degree of shading produced by different quantities of foliage and the response of foliage to different light levels (this information is derived from literature values, field measurements, or simulation models). A comparable but simpler set of data on minor vegetation must be provided if the user wishes to represent this important ecosystem component (e.g., Royo and Carson 2006). Data are obtained from the literature or field measurements. Lastly, data describing the rates of decomposition of various litter types and soil organic matter are required for the model to simulate nutrient cycling. A detailed description of the input data requirements can be found in Kimmins and others (1999), or from the corresponding author.

With the calibration data obtained from different sources, the model calculates the annual rates of different ecological processes (tree growth, litterfall production, mortality, etc.) based on the

historical data on tree growth and density provided by the user. Therefore, for each plant species for which historical data are provided, the total net primary production (TNPP) that occurred for each annual time step (t) is calculated with Eq. 3.

$$TNPP_t = \Delta biomass_t + litterfall_t + mortality_t \quad (3)$$

where $\Delta biomass_t$ = the sum of the change in mass of all the biomass components of the particular species in time step *t*; $litterfall_t$ = the sum of the mass of all ephemeral tissues that are lost in time step t (e.g., leaf, branch, bark and reproductive litterfall, and root death), and $mortality_t$ = the mass of individual plants that die in time step *t*. Change in biomass ($\Delta biomass_t$) in each time step is derived from a series of age–biomass curves created with empirical data. Litterfall is calculated using user-defined values based on empirical litterfall rates. Mortality is derived from a series of age–stand density curves created with empirical data (for a detailed description on mortality simulation in FORECAST, see Kimmins *et al.* 1999). Mortality is calibrated through two different parameters: curves of historical stand density for different ages and the proportion of mortality that is due to non-intraspecific competition factors.

The model also estimates the shade-corrected foliage N content (SCFN), which represents the amount of fully illuminated foliage N that was required to produce the calculated historical TNPP. To estimate foliage shading, FORECAST simulates canopy foliage biomass as a "blanket" that covers the stand and that is divided in several layers of 0.25 m height, each of them increasingly darker from the top to the bottom of the canopy. The light absorbed by each layer is calculated based on the foliage biomass present in each time step and a user-defined empirical curve of foliage mass-proportion of full light. Once an estimation of self-shading has been completed for a particular time step using the method described above, FORECAST calculates a foliar N content adjusted for the effects of self-shading (Eq. 4 and 5).

$$SCFN_t = \sum_{i=1}^{n}(FN_{t,i} \times PLSC_i) \quad (4)$$

$$FN_{t,i} = \text{foliage biomass}_{t,i} \text{ x foliar N concentration} \quad (5)$$

where $FN_{t,i}$ = mass of foliage nitrogen in the *ith* quarter-meter height increment in the live canopy at time *t*, $PLSC_i$ = photosynthetic light saturation curve value for the associated light level in the *ith* quarter-meter height increment in the live canopy, n = number of quarter-meter height increments in the live canopy at time *t*. The mean photosynthetic rate of the foliage in canopy level *i* is calculated by combining simulated light intensities in canopy level *i* with input data that define photosynthetic light saturation curves for the foliage type in question. Finally, the driving function curve for potential growth of a given species in FORECAST is the shade-corrected foliar nitrogen efficiency (SCFNE) calculated for each annual time step (*t*) with Eq. 6:

$$SCFNE_t = TNPP_t / SCFN_t \quad (6)$$

When data describing the growth of a species on more than one site quality (i.e. nutrient availability) are provided, SCNFE function curves will be generated during the calibration stage for each site quality. To calculate the nutritional aspects of tree and plant growth, FORECAST requires data on nutrient concentration in each different tree organ. Nutrient dynamics in this study were restricted to nitrogen (the most limiting nutrient at this region (Wang *et al.* 2013, Bi *et al.* 2007, Blanco *et al.* 2012, Wei *et al.* 2012).

The combination of light and nutrient limitation is usually not enough to explain complex ecological patterns through models, and also including understory vegetation in the simulations is recommended (Kimmins *et al.* 2008). Therefore, a comparable but simpler (e.g. no data on bark, wood, mortality, etc.) set of data for understory vegetation must be provided to represent this ecosystem component. Lastly, data describing decomposition rates for various litter and humus types are required to simulate nutrient cycling. Decomposition rates are defined by the user (using values from empirical studies) and are affected by site quality, which in turn is defined depending on nutrient and water availability. Snags and logs are tracked by placing them into different categories depending on their original sizes (with slower decomposition rates for snags and for stems with larger sizes).

The second stage of calibration requires running the model in "set-up" mode to establish initial site conditions. In this stage, the model is run with nutrient feedback turned off to allow it to accumulate vegetation, litter and soil organic matter representative of the site(s) to be modeled, and which reflects the historical patterns of accumulation. This is typically achieved by simulating the

known or estimated natural disturbance and/or management history of the site (see Seely and others 2002; or Blanco and others 2007 for a detailed description of this process).

After calibrating, estimating the historical ecological rates, and creating the initial conditions, the model is ready to simulate each particular scenario. During the simulation stage, for each annual time step, the annual potential growth (APG) of vegetation is driven by the photosynthetic production of the foliage biomass (Eq. 7). The productive capacity of a given quantity of foliage biomass (photosynthetic rate) is assumed to be dependent on foliage nitrogen content corrected for shading created by the canopy of the simulated site ($SCFN_t$*). $SCFN_t$* is different from the $SCFN_t$ that was previously calculated during the internal calibration stage. During the simulation stage the canopy simulated corresponds to the site defined by the user for that particular scenario, which can be different from the empirical canopy data used (i.e. different stand density) during the calibration stage, and therefore $SCFN_t$* is particular for each simulation.

$$APG_{(t+1)} = SCFN_t^* \text{ x } SCFNE_t \qquad (7)$$

where: $APG_{(t+1)}$ = annual potential growth for a given species in the next time step. During the simulation stage, the model interpolates between the different curves of SCFNE calculated before to find the site quality of the simulated site. Nutrient uptake requirements to support APG are calculated based on rates of biomass growth and data on nutrient concentration in the different biomass components. Nutrient availability is calculated based on empirical data describing litter and humus decomposition rates, changes in chemistry as decomposition proceeds, and the size of nutrient pools in the mineral soil and humus (cation exchange capacity (CEC) and anion exchange capacity (AEC), respectively). If the availability of nutrients for each time step is less than required to support APG, vegetation growth is limited by nutrients and the realized annual growth is lower than APG.

Nitrogen cycling in FORECAST is based on a mass balance approach (Figure 4) where N can exist in three distinct pools: 1) the plant biomass pool; 2) the available soil nutrient pool, and 3) the soil organic matter/forest floor pool. Inputs and outputs of N to the ecosystem are simulated in a four-stage process for each annual time step. The “available N” pool in FORECAST can be assimilated to represent the interchangeable N present in the soil during one year as NH_4^+ , NO_3^- or labile organic N

fractions with turnover rates shorter than one year. N deposition and N fixed by bryophytes and other microorganisms are simulated as constant annual N fluxes that directly reach the soil solution and are incorporated into the available N pool. Annual values of available N are calculated by simulating consecutively the different inputs and outputs of the biogeochemical cycle: deposition, fertilization, seepage, leaching, mineralization, immobilization (Figure 4). The simulation of each of these fluxes in FORECAST has been described in detail before *(*Kimmins *et al.* 1999, Blanco *et al.* 2012). The definition of site fertility based on N availability assumes that soil moisture is not limiting in these sites (Blanco *et al.* 2012, Wei *et al.* 2012, Wang *et al.* 2013). However, soil moisture is still implicitly affecting the simulation by the use of the parameter "maximum foliage per tree" which is directly correlated with soil moisture availability (Kimmins *et al.* 1999).

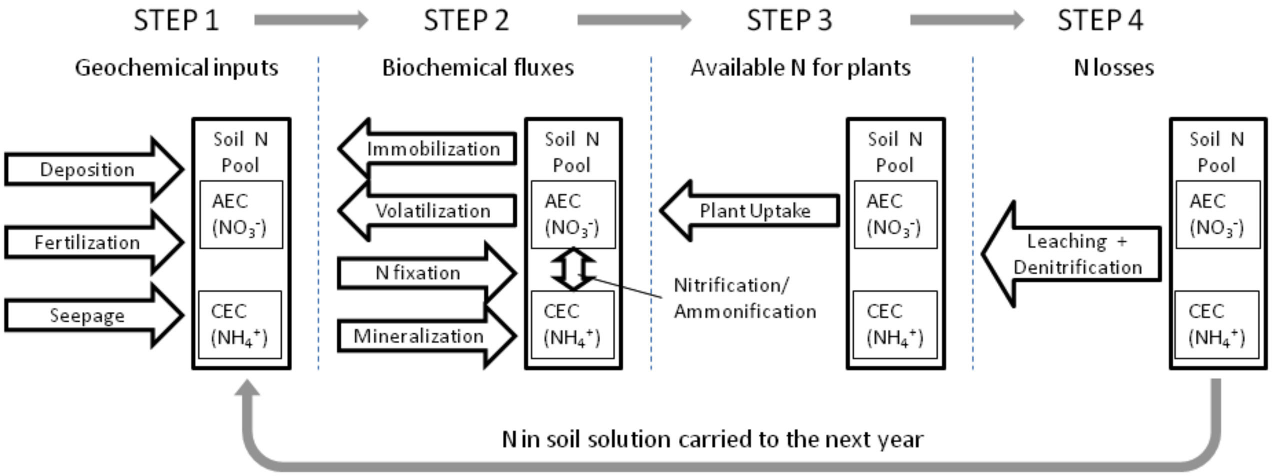

Figure S2. Estimation of available N in FORECAST in each annual time step. Step 1: geochemical inputs were calculated, with all the forms of N lumped together. Step 2: biochemical fluxes. Step 3: Plants uptake the available N. Step 4: Soil N remaining for next time step is calculated by subtracting the remaining N from the soil CEC (for ammonium) or AEC (for nitrate). The N excess was assumed to be lost via leaching.

Carbon and nitrogen cycles are linked through the use of the foliar N efficiency as the driving function of the model (amount of biomass generated in a year per kg of foliar N). Therefore, a limitation in N uptake will result in a reduction of foliar N, reducing biomass produced by the trees.

Nutrient uptake demands on sites of different N fertility are based on observed biomass accumulation rates and tissue nutrient concentrations on these sites, allowing for internal cycling of nutrients.

3. FORECAST model evaluation for Chinese fir and *Phoebe bournei* plantations

Published field data on several chronosequences at different site qualities were used to evaluate FORECAST performance for Chinese fir plantations in SE China (Tian 2003, Rong et al. 2008). Data to evaluate FORECAST performance for *Phoebe bournei* plantations were obtained from literature(Ma et al. 2008, Peng 2003, Peng 2008a, Peng 2008b, Wu 2009, Liao et al. 1989, Chen et al. 2007, Cai 2009, Liang et al. 2009 Li 2003, Long et al. 2011, sun 2008, tong 2010, Wei an Ma 2006, Zhang and Wu 2007). For our purposes we used the data described for a good site (27 m dominant height at stand age 50 years) and in areas with low levels of N deposition (5 kg ha^{-1} y^{-1}), assuming that this is the historical level of N deposition in which the forests described by Tian (2003) and Rong *et al.* (2008) grew (Wei et al 2012) To assess the performance of FORECAST relative to field observations, data pairs of observed vs. predicted were subjected to graphical comparisons, assessments of average and absolute biases, and measures of goodness-of-fit (Blanco et al. 2007). A linear regression of predicted vs. observed values was fitted to calculate the coefficient of determination (r^2). In addition, two different indices were calculated.

The first performance index was Theil's inequality coefficient (Theil 1966):

$$U = \sqrt{\frac{\sum_{i=q}^{n} D_i}{\sum_{i=1}^{n} Observed_i^2}} \qquad (6)$$

where D_i is the difference between $Observed_i$ and $Predicted_{i,}$ and n is the number of data pairs. U can assume values of 0 and greater. If U = 0 then the model produces perfect predictions. If U = 1 the model produces predictions of system behaviour that are not better than assuming the system does not change. If U > 1, then the predictive power of the model is worse than the no-change prediction. The second index was modelling efficiency (ME) (Vanclay an Skovsgaard 1997):

$$ME = 1 - \frac{\sum D_i^2}{\sum (Observed_i - \overline{predicted})^2} \quad (7)$$

This statistic provides a simple index of performance on a relative scale, where ME = 1 indicates a perfect fit, ME = 0 reveals that the model is no better than a simple average, while negative values indicate poor performance. Finally, the critical error e* was calculated for two different confidence levels[64]. This error can be interpreted as the smallest error level, in absolute terms, which will lead to the acceptance of the null hypothesis (i.e. that the model is within e* units of the true value). If e* is lower than the accuracy level defined by the model user (the minimum acceptable difference between observed and modelled values), then the model is accepted as suitable for the model user's needs. All statistical analyses were carried out using JMP version 5.0.1.2 from SAS Institute.

All the indices of model performance indicated that FORECAST produced acceptable predictions (Table S1), although the results were better for Chinese fir than for *Phoebe bournei*. Model predictions were better for the first half of the rotation (until year 25), after which there was a slight tendency to underestimate DBH, aboveground biomass and forest litter mass (Figure S4). Critical error values were low, less than 10% of the maximum value of all variables except forest floor litter mass, for which the values were 20% and 16% of the maximum mass for confidence levels of 95% and 80%, respectively.

Table S1. Indices of FORECAST performance for simulations of four variables compared with field data for Chinese fir (CF) and *Phoebe bournei* (PB). e*: critical error at two different levels of confidence (95% and 80%).

Measurement of model performance	Top height (m)		Dominant DBH (cm)		Aboveground biomass (Mg ha^{-1})		Forest floor litter mass[a] (Mg ha^{-1})
	CF	PB	CF	PB	CF	PB	CF
Average bias	0.66	-0.55	-0.47	-0.31	-5.89	1.40	0.01
Mean absolute deviation	1.01	2.71	0.98	3.26	11.38	24.75	0.59
Pearson's *r*	0.98	0.81	0.98	0.80	0.97	0.80	0.91
Theil's inequality coefficient	0.07	0.22	0.07	0.24	0.13	0.21	0.21
Modelling efficiency	0.96	0.96	0.92	0.95	0.95	0.94	0.85
Relaxed e* (α = 0.05)	1.63	5.52	1.69	6.91	21.91	45.40	1.03
Exigent e* (α = 0.20)	1.23	3.96	1.27	4.94	15.94	33.34	0.78

[a] Forest floor data available only for Chinese fir plantations.

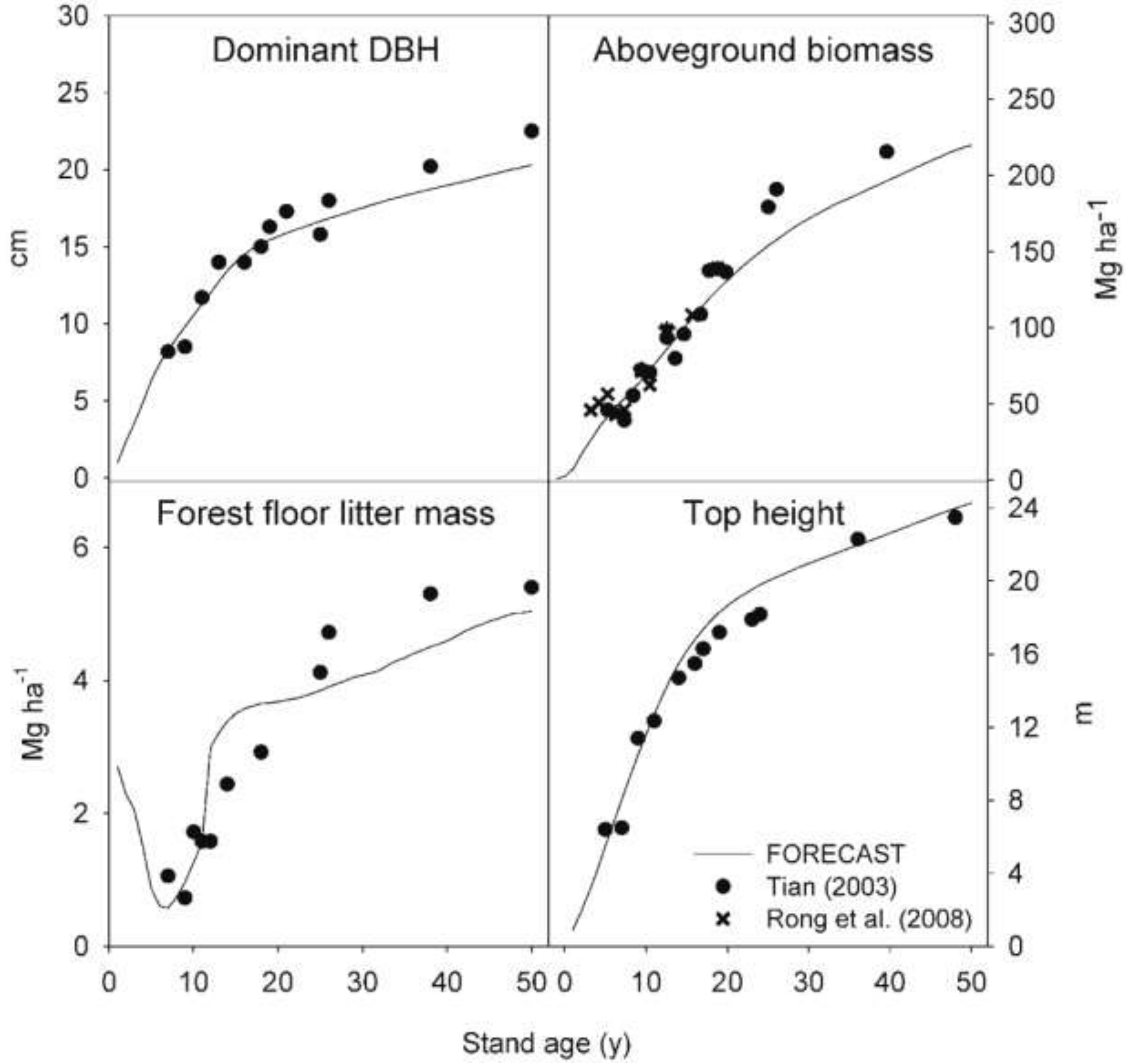

Figure S3. Comparison between simulated values of four variables and field values reported by Tian (2003) and Rong *et al.* (2008)for a Chinese fir plantation in SE China with site index 27 m at year 50 in areas with low levels of N deposition (5 kg ha^{-1} y^{-1}).

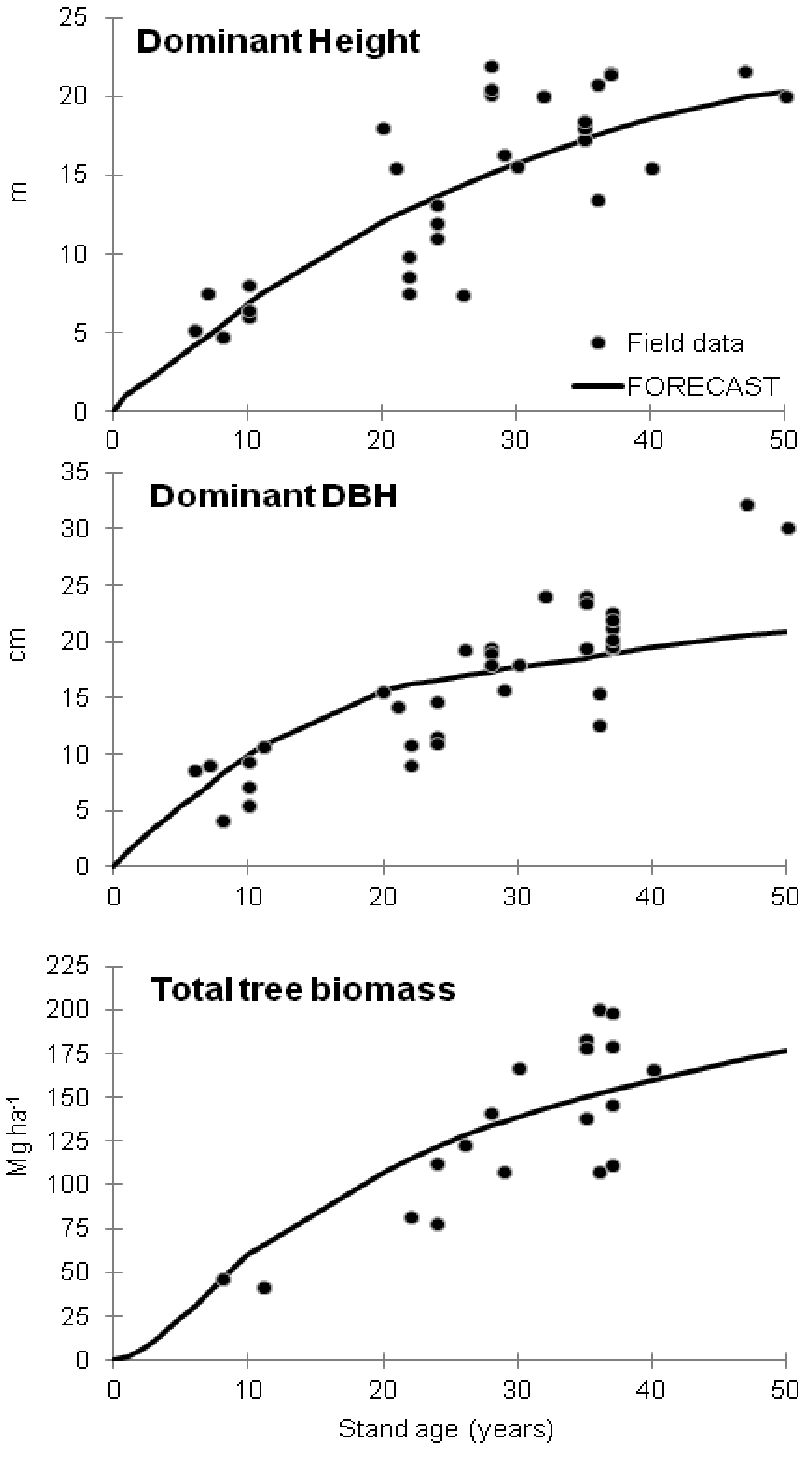

Figure S4. Comparison between simulated values of four variables and field values reported by for a *Phoebe bournei* plantation in SE China with site index 27 m at year 50 in areas with low levels of N deposition (5 kg ha^{-1} y^{-1}).

Low average biases and absolute differences, together with high Pearson's *r* values, indicate acceptable agreement between observed and predicted values. However, it has been argued that *r* is not the most reliable measure of model performance (Power 1993) because it is not related to the "perfect fit" line (the line in which observed equals predicted). As a consequence, this coefficient is more about a model's capacity to use the calibration data set to reduce differences between observed and predicted values rather than a measure of the accuracy of a model's predictions. A different measure of model performance is given by Theil's U coefficient, whose values were always lower than 1, indicating that the model always performed better than a general average value such as that provided by traditional growth and yield tables. Modelling efficiency, recommended as a more adequate measure of model performance (Power 1993, Mayer and Butler 1993, Smith et al. 1997) was close to 1 for all variables, indicating acceptable agreement between observed and simulated values.

Finally, Reynolds' critical values were low, showing that FORECAST is capable of meeting the requirements of users who need high levels of accuracy. FORECAST is an ecosystem-level model that integrates key ecosystem processes with field observations, with a hybrid approach that provides robustness to the predictions and improves the ecological performance of the model (Kimmins et al 1999, Kimmins et al. 2010, Blanco et al. 2007). The acceptable results of predictions from this study are evidence of this ecological performance. These positive evaluation results are in agreement with the performance of FORECAST in other similar subtropical Chinese fir forests (Bi et al. 2007), temperate and tropical plantations (Blanco et al. 2007, Blanco and González 2010) and boreal natural forests (Seely et al. 2008). Therefore, FORECAST appears to be a valuable tool for studying ecological processes in forest ecosystems in situations when robust predictions are needed.

4. References used for the review analysis

The following tables provide the details of all the studies used to estimate carbon pools in subtropical plantations.

Table S2. List of sites used to calculate the range of belowground, aboveground and ecosystem C in tropical broadleaf plantations. Belowground C has been standardized at soil depth 60 cm (see main text).

Country	Species	Age<br>Years	tree density<br>Stems ha^{-1}	Above. C<br>Mg C ha^{-1}	Below. C<br>Mg C ha^{-1}	Ecosys. C<br>Mg C ha^{-1}	Reference
Vietnam	Acacia sp.	-	-	32.10	56.82	88.92	Sang et al. (2013)
Vietnam	Acacia sp.	-	-	49.20	65.12	114.32	Sang et al. (2013)
China	*Castanopsis kawakamii*	33.0	-	161.52	135.59	296.37	Zheng et al. (2009)
Ghana	Cocoa tree	21.0	1098	65.00	-	-	Kongsager et al. (in press)
China	Cucumis hystrix	25.0	415	-	71.09	-	Wang et al. (in press)
China	Eucalyptus spp.	-	-	-	76.14	-	Zheng et al. (2009)
Ethiopia	Eucalyptus spp.	22.0	500	211.14	234.39	445.53	Demessie et al. (2011)
Ethiopia	Eucalyptus spp.	22.0	600	240.24	250.95	491.19	Demessie et al. (2011)
Ethiopia	Eucalyptus spp.	30.0	750	370.38	272.89	643.27	Demessie et al. (2011)
India	Eucalyptus spp.	-	-	41.00	67.00	108.00	Kaul et al. (2010)
Madagascar	Eucalyptus spp.	5.0	-	28.00	60.43	103.43	Razakamanarivo et al. (2011)
Madagascar	Eucalyptus spp.	5.0	-	77.00	108.43	189.43	Razakamanarivo et al. (2011)
South Africa	Eucalyptus spp.	25.0	-	269.90	-	-	Christie and Scholes (1995)
South Africa	Eucalyptus spp.	10.0	-	47.40	-	-	Christie and Scholes (1995)
Vietnam	Eucalyptus spp.	-	-	29.30	52.92	82.22	Sang et al. (2013)
Vietnam	Eucalyptus spp.	-	-	42.70	54.72	97.42	Sang et al. (2013)
Costa Rica	*Gmelina arborea*	17.0	1200	69.23	-	-	Arias et al. (2011)
Costa Rica	*Hieronyma alchorneoides*	3.2	1089	3.30	86.02	89.32	Fonseca et al. (2012)
Costa Rica	*Hieronyma alchorneoides*	7.2	1089	6.70	85.62	92.32	Fonseca et al. (2012)
Costa Rica	*Hieronyma alchorneoides*	5.0	1089	17.30	94.02	111.32	Fonseca et al. (2012)
Costa Rica	*Hieronyma alchorneoides*	9.1	1089	42.10	127.32	169.42	Fonseca et al. (2012)
Costa Rica	*Hieronyma alchorneoides*	14.7	1089	42.40	138.22	180.62	Fonseca et al. (2012)
Costa Rica	*Hieronyma alchorneoides*	16.0	1089	63.70	117.12	180.82	Fonseca et al. (2012)
Costa Rica	*Hieronyma alchorneoides*	12.0	1089	47.20	207.72	254.92	Fonseca et al. (2012)
DR Congo	Limba	54.0	104	54.20	-	-	De Ridder et al. (2010)
China	*Michelia macclurei*	10.0	-	22.79	-	120.87	Wang et al. (in press)
China	*Michelia macclurei*	24.0	2000	56.79	75.60	132.39	Wang et al. (2007b)
China	*Michelia macclurei*	22.0	975	63.50	84.97	148.47	Niu et al. (2009)
China	*Michelia macclurei*	22.0	-	64.39	94.82	159.21	Wang et al. (in press)
China	*Michelia macclurei*	20.0	2000	56.79	116.47	173.26	Huang et al. (2005)
China	*Michelia macclurei*	20.0	2000	71.00	116.47	187.47	Huang et al. (2004)
China	*Michelia macclurei*	22.0	975	75.00	70.27	145.27	Niu et al. (2009)
China	Moso bamboo	14.0	3500	41.41	50.03	91.44	Jiang et al. (2011)
Taiwan	Moso bamboo	5.0	7078	40.60	-	-	Yen et al. (2011)
China	Nanmu	8.0	1985	27.00	69.26	96.26	Wei and Ma (2006)
China	Nanmu	24.0	1360	63.40	73.45	136.85	Wei and Ma (2006)

Country	Species	Age	tree density	Above. C	Below. C	Ecosys. C	Reference
China	Nanmu	35.0	832	98.01	82.78	180.79	Wei and Ma (2006)
China	Nanmu	32.0	3333	94.05	112.16	205.11	Ma et al. (2009)
China	Nanmu	26.0	1100	63.20	-	-	Cai (2009)
Australia	Native broadleaf species	12.0	-	50.87	-	-	Kanowski and Catterall (2010)
Australia	Native broadleaf species	12.0	-	70.70	-	-	Kanowski and Catterall (2010)
Australia	Native broadleaf species	14.0	-	78.30	-	-	Kanowski and Catterall (2010)
Australia	Native broadleaf species	14.0	-	98.70	-	-	Kanowski and Catterall (2010)
Australia	Native broadleaf species	14.0	-	117.60	-	-	Kanowski and Catterall (2010)
China	Native broadleaf species	25.0	400	-	73.34	-	Wang et al. (in press)
Costa Rica	Native broadleaf species	9.5	712	91.00	-	-	Redondo-Brenes (2007)
Costa Rica	Native broadleaf species	11.5	692	36.90	-	-	Redondo-Brenes (2007)
Costa Rica	Native broadleaf species	9.5	576	79.10	-	-	Redondo-Brenes (2007)
Costa Rica	Native broadleaf species	11.5	385	55.10	-	-	Redondo-Brenes (2007)
Costa Rica	Native broadleaf species	9.5	563	66.30	-	-	Redondo-Brenes (2007)
Costa Rica	Native broadleaf species	11.5	338	46.50	-	-	Redondo-Brenes (2007)
Costa Rica	Native broadleaf species	9.5	545	44.50	-	-	Redondo-Brenes (2007)
Costa Rica	Native broadleaf species	11.5	269	41.20	-	-	Redondo-Brenes (2007)
Costa Rica	Native broadleaf species	9.5	353	23.50	-	-	Redondo-Brenes (2007)
Costa Rica	Native broadleaf species	11.5	280	36.30	-	-	Redondo-Brenes (2007)
Costa Rica	Native broadleaf species	11.5	436	22.50	-	-	Redondo-Brenes (2007)
Costa Rica	Native broadleaf species	11.5	684	60.00	-	-	Redondo-Brenes (2007)
Costa Rica	Native broadleaf species	9.5	688	36.50	-	-	Redondo-Brenes (2007)
Costa Rica	Native broadleaf species	13.5	852	44.40	-	-	Redondo-Brenes (2007)
Costa Rica	Native broadleaf species	9.5	411	27.50	-	-	Redondo-Brenes (2007)
Costa Rica	Native broadleaf species	9.5	552	28.30	-	-	Redondo-Brenes (2007)
Costa Rica	Native broadleaf species	11.5	680	28.80	-	-	Redondo-Brenes (2007)
Costa Rica	Native broadleaf species	9.5	498	36.60	-	-	Redondo-Brenes (2007)
Costa Rica	Native broadleaf species	11.5	654	39.70	-	-	Redondo-Brenes (2007)
Costa Rica	Native broadleaf species	13.5	711	40.50	-	-	Redondo-Brenes (2007)
Costa Rica	Native broadleaf species	9.5	434	23.00	-	-	Redondo-Brenes (2007)
Costa Rica	Native broadleaf species	11.5	587	20.00	-	-	Redondo-Brenes (2007)
Costa Rica	Native broadleaf species	9.5	400	12.40	-	-	Redondo-Brenes (2007)
Costa Rica	Native broadleaf species	13.5	817	21.20	-	-	Redondo-Brenes (2007)
Mexico	Native broadleaf species	-	-	50.60	98.22	148.82	Ordoñez et al. (2008)
Panama	Native broadleaf species	8.0	700	5.44	-	-	Ruiz-Jaen and Potvin (2011)
Puerto Rico	Native broadleaf species	70.0	3762	80.00	92.61	172.61	Silver et al. (2004)
Ghana	Oil palm	10.0	144	21.70	-	-	Kongsager et al. (in press)
Ghana	Oil palm	16.0	144	28.00	-	-	Kongsager et al. (in press)
Ghana	Oil palm	23.0	144	45.30	-	-	Kongsager et al. (in press)
Ghana	Orange tree	25.0	267	76.30	-	-	Kongsager et al. (in press)
China	Poplar spp.	7.0	1500	5.44	15.95	21.39	Hu et al. (2008)
China	Poplar spp.	11.0	1333	20.43	14.29	34.72	Hu et al. (2008)

Country	Species	Age	tree density	Above. C	Below. C	Ecosys. C	Reference
China	Poplar spp.	15.0	1289	43.98	21.53	65.51	Hu et al. (2008)
China	Poplar spp.	4.0	1111	23.50	-	-	Fang et al. (2007)
China	Poplar spp.	4.0	833	20.70	-	-	Fang et al. (2007)
China	Poplar spp.	4.0	625	18.10	-	-	Fang et al. (2007)
China	Poplar spp.	4.0	500	13.70	-	-	Fang et al. (2007)
China	Poplar spp.	6.0	1111	39.50	-	-	Fang et al. (2007)
China	Poplar spp.	6.0	833	35.20	-	-	Fang et al. (2007)
China	Poplar spp.	6.0	625	32.30	-	-	Fang et al. (2007)
China	Poplar spp.	6.0	500	26.80	-	-	Fang et al. (2007)
China	Poplar spp.	8.0	1111	53.30	-	-	Fang et al. (2007)
China	Poplar spp.	8.0	833	47.10	-	-	Fang et al. (2007)
China	Poplar spp.	8.0	625	44.60	-	-	Fang et al. (2007)
China	Poplar spp.	8.0	500	38.30	-	-	Fang et al. (2007)
China	Poplar spp.	10.0	1111	62.50	-	-	Fang et al. (2007)
China	Poplar spp.	10.0	833	58.70	-	-	Fang et al. (2007)
China	Poplar spp.	10.0	625	54.70	-	-	Fang et al. (2007)
China	Poplar spp.	10.0	500	48.10	-	-	Fang et al. (2007)
India	Poplar spp.	-	-	55.00	75.00	130.00	Kaul et al. (2010)
India	Poplar spp.	8.0	500	96.23	-	-	Sing and Lohiyal (2009)
Mexico	Poplar spp.	19.0	1252	5.51	-	-	Rios-Carrasco et al. (2009)
Mexico	Poplar spp.	19.0	2071	9.29	-	-	Rios-Carrasco et al. (2009)
Mexico	Poplar spp.	19.0	3373	11.10	-	-	Rios-Carrasco et al. (2009)
Mexico	Poplar spp.	19.0	3973	17.00	-	-	Rios-Carrasco et al. (2009)
Mexico	Poplar spp.	19.0	4163	20.70	-	-	Rios-Carrasco et al. (2009)
Mexico	Poplar spp.	19.0	5919	32.26	-	-	Rios-Carrasco et al. (2009)
Myanmar	*Pterocarpus macrocarpus*	15.0	-	-	64.07	124.19	Aye et al. (2011)
Brazil	Rubber tree	14.0	-	41.70	88.06	139.61	Wauters et al. (2008)
Ghana	Rubber tree	12.0	-	61.50	-	-	Kongsager et al. (in press)
Ghana	Rubber tree	44.0	-	213.60	-	-	Kongsager et al. (in press)
Ghana	Rubber tree	14.0	-	76.30	39.03	121.61	Wauters et al. (2008)
China	Tea oil tree	14.0	2433	21.10	41.47	82.47	Zheng et al. (2008)
Colombia	Teak	8.0	997	47.46	45.55	93.01	Loaiza et al. (2010)
Colombia	Teak	13.5	1268.5	130.66	72.96	203.62	Loaiza et al. (2010)
Colombia	Teak	21.5	1540	213.44	112.40	325.85	Loaiza et al. (2010)
Ghana	Teak	14.0	3333	38.00	78.36	89.36	Watanabe et al. (2009)
Ghana	Teak	14.0	3333	36.90	81.36	96.56	Watanabe et al. (2009)
Ghana	Teak	14.0	3333	48.70	67.16	120.76	Watanabe et al. (2009)
Ghana	Teak	14.0	3333	50.80	70.36	131.46	Watanabe et al. (2009)
Ghana	Teak	14.0	3333	66.00	105.66	154.36	Watanabe et al. (2009)
Ghana	Teak	14.0	3333	15.20	97.66	156.76	Watanabe et al. (2009)
Ghana	Teak	14.0	3333	72.00	85.96	171.96	Watanabe et al. (2009)
Ghana	Teak	14.0	3333	23.10	149.16	177.46	Watanabe et al. (2009)

Country	Species	Age	tree density	Above. C	Below. C	Ecosys. C	Reference
Ghana	Teak	14.0	3333	27.20	120.56	277.26	Watanabe et al. (2009)
India	Teak	-	-	50.00	52.00	102.00	Kaul et al. (2010)
Panama	Teak	10.0	-	12.10	-	30.70	Potvin et al. (2004)
Panama	Teak	-	-	120.00	225.00	351.00	Kraenzel et al. (2003)
Panama	Teak	1.0	1079	2.90	-	-	Derwish et al. (2009)
Panama	Teak	2.0	990	6.60	-	-	Derwish et al. (2009)
Panama	Teak	10.0	383	40.70	-	-	Derwish et al. (2009)
Cameroon	*Terminalia ivorensis*	9.0	192	71.20	-	-	Derwish et al. (2009)
Cameroon	*Terminalia ivorensis*	20.0	192	84.10	-	-	Derwish et al. (2009)
Costa Rica	*Vochysia guatemalensis*	0.7	1089	2.74	94.32	97.06	Fonseca et al. (2012)
Costa Rica	*Vochysia guatemalensis*	0.0	1089	1.10	124.92	126.02	Fonseca et al. (2012)
Costa Rica	*Vochysia guatemalensis*	3.2	1089	7.30	122.62	129.92	Fonseca et al. (2012)
Costa Rica	*Vochysia guatemalensis*	7.0	1089	37.10	119.12	156.22	Fonseca et al. (2012)
Costa Rica	*Vochysia guatemalensis*	5.0	1089	27.20	134.62	161.82	Fonseca et al. (2012)
Costa Rica	*Vochysia guatemalensis*	14.0	1089	60.20	116.92	177.12	Fonseca et al. (2012)
Costa Rica	*Vochysia guatemalensis*	16.0	1089	69.80	142.92	212.72	Fonseca et al. (2012)
Costa Rica	*Vochysia guatemalensis*	9.0	1089	47.60	166.72	214.32	Fonseca et al. (2012)
Myanmar	*Xylia xylocarpa*	15.0	-	-	52.15	112.86	Aye et al. (2011)

Table S3. List of sites used to calculate the range of belowground, aboveground and ecosystem C in tropical conifer plantations. Belowground C has been standardized at soil depth 60 cm (see main text).

Country	Species	Age	Tree density	Above. C	Below. C	Ecosys. C	Reference
		Years	Stems ha^{-1}	Mg C ha^{-1}	Mg C ha^{-1}	Mg C ha^{-1}	
Australia	Araucaria	11.0	-	60.00	-	-	Kenowski and Catterall (2010)
Australia	Araucaria	12.0	-	49.67	-	-	Kenowski and Catterall (2010)
Australia	Araucaria	12.0	-	58.70	-	-	Kenowski and Catterall (2010)
Australia	Araucaria	14.0	-	53.00	-	-	Kenowski and Catterall (2010)
Australia	Araucaria	16.0	-	74.50	-	-	Kenowski and Catterall (2010)
China	Chinese fir	2.0	5210	9.30	59.47	70.47	Chen et al. (in press)
China	Chinese fir	7.0	4892	33.00	44.47	80.47	Chen et al. (in press)
China	Chinese fir	0.0	0	0.00	85.47	85.47	Chen et al. (in press)
China	Chinese fir	16.0	3875	61.00	35.47	100.47	Chen et al. (in press)
China	Chinese fir	21.0	2800	82.00	45.47	125.47	Chen et al. (in press)
China	Chinese fir	-	-	-	84.48	-	Du et al. (2001)
China	Chinese fir	-	-	-	72.18	-	Du et al. (2001)
China	Chinese fir	-	-	-	43.55	-	Fang (1987)
China	Chinese fir	-	-	-	38.04	-	Fang (1987)
China	Chinese fir	-	-	-	52.83	-	Fang (1987)

Country	Species	Age	Tree density	Above. C	Below. C	Ecosys. C	Reference
China	Chinese fir	22.0	-	-	94.61	-	Fang et al. (2006)
China	Chinese fir	22.0	-	-	66.90	-	Fang et al. (2006)
China	Chinese fir	22.0	-	-	76.54	-	Fang et al. (2006)
China	Chinese fir	27.0	2080	30.50	67.07	97.57	Fang et al. (2002
China	Chinese fir	27.0	1650	-	77.47	-	Guo et al. (2006)
China	Chinese fir	12.0	-	47.20	-	-	He and Yu (1992)
China	Chinese fir	14.0	-	39.30	-	-	He and Yu (1992)
China	Chinese fir	17.0	-	84.10	-	-	He and Yu (1992)
China	Chinese fir	17.0	-	45.80	-	-	He and Yu (1992)
China	Chinese fir	21.0	2000	-	109.04	-	He et al. (2007)
China	Chinese fir	21.0	2000	-	80.64	-	He et al. (2007)
China	Chinese fir	15.0	2500	69.90	97.63	167.53	Huang et al. (in press)
China	Chinese fir	15.0	2500	80.40	103.43	183.83	Huang et al. (in press)
China	Chinese fir	15.0	2500	79.40	104.53	183.93	Huang et al. (in press)
China	Chinese fir	15.0	2500	79.70	109.33	189.03	Huang et al. (in press)
China	Chinese fir	20.0	2000	70.50	133.47	203.97	Huang et al. (2004)
China	Chinese fir	20.0	2000	97.34	133.47	230.81	Huang et al. (2005)
China	Chinese fir	14.0	3100	61.55	20.72	82.27	Jiang et al. (2010)
China	Chinese fir	20.0	-	-	41.87	-	Lin et al. (1992)
China	Chinese fir	20.0	-	-	35.27	-	Lin et al. (1992)
China	Chinese fir	18.0	2200	-	142.38	-	Luan et al. (2010)
China	Chinese fir	6.0	3350	23.90	60.48	109.79	Ma et al. (2010)
China	Chinese fir	9.0	2195	33.00	54.00	111.98	Ma et al. (2010)
China	Chinese fir	16.0	1650	47.40	45.86	117.69	Ma et al. (2010)
China	Chinese fir	6.0	3320	32.30	62.98	120.86	Ma et al. (2010)
China	Chinese fir	9.0	2174	44.20	58.92	128.43	Ma et al. (2010)
China	Chinese fir	5.0	3340	41.20	62.41	129.15	Ma et al. (2010)
China	Chinese fir	8.0	2300	57.90	58.36	141.53	Ma et al. (2010)
China	Chinese fir	22.0	1800	55.70	72.38	152.14	Ma et al. (2010)
China	Chinese fir	15.0	1550	73.40	54.00	152.38	Ma et al. (2010)
China	Chinese fir	15.0	1575	63.90	65.22	154.85	Ma et al. (2010)
China	Chinese fir	19.0	1550	71.80	70.37	168.24	Ma et al. (2010)
China	Chinese fir	19.0	1566	90.50	55.69	171.28	Ma et al. (2010)
China	Chinese fir	6.0	3225	25.00	34.59	59.59	Ma et al. (2002)
China	Chinese fir	22.0	940	59.80	64.67	124.47	Huang et al. (2005)
China	Chinese fir	22.0	940	69.10	52.74	121.84	Huang et al. (2005)
China	Chinese fir	20.0	-	-	93.08	-	Shao (1992)
China	Chinese fir	20.0	-	-	88.60	-	Shao (1992)
China	Chinese fir	20.0	-	-	93.78	-	Shao (1992)
China	Chinese fir	20.0	-	-	72.34	-	Shao (1992)
China	Chinese fir	20.0	-	-	70.15	-	Shao (1992)
China	Chinese fir	20.0	-	-	99.07	-	Shao (1992)

Country	Species	Age	Tree density	Above. C	Below. C	Ecosys. C	Reference
China	Chinese fir	20.0	-	-	84.26	-	Shao (1992)
China	Chinese fir	20.0	-	-	90.52	-	Shao (1992)
China	Chinese fir	20.0	-	-	99.84	-	Shao (1992)
China	Chinese fir	20.0	-	-	85.97	-	Shao (1992)
China	Chinese fir	20.0	-	-	55.34	-	Shao (1992)
China	Chinese fir	40.0	-	-	71.21	-	Shao et al. (2007)
China	Chinese fir	12.0	-	-	196.68	-	Shao et al. (2007)
China	Chinese fir	8.0	-	-	87.52	-	Shao et al. (2007)
China	Chinese fir	16.0	-	-	58.47	-	Shao et al. (2007)
China	Chinese fir	10.0	-	-	89.47	-	Shao et al. (2007)
China	Chinese fir	6.0	3267	5.60	59.37	68.40	Sun et al. (2003)
China	Chinese fir	6.0	2500	5.90	62.92	72.48	Sun et al. (2003)
China	Chinese fir	12.0	1800	19.80	58.68	91.78	Sun et al. (2003)
China	Chinese fir	6.0	1725	19.80	72.28	96.37	Sun et al. (2003)
China	Chinese fir	5.0	3867	33.50	60.51	97.51	Sun et al. (2003)
China	Chinese fir	4.0	2300	20.70	75.77	100.99	Sun et al. (2003)
China	Chinese fir	12.0	3900	34.70	67.98	106.68	Sun et al. (2003)
China	Chinese fir	12.0	2600	54.30	67.98	116.51	Sun et al. (2003)
China	Chinese fir	12.0	-	32.70	91.87	120.65	Sun et al. (2003)
China	Chinese fir	12.0	3850	51.90	58.82	124.21	Sun et al. (2003)
China	Chinese fir	12.0	2233	49.10	94.07	146.56	Sun et al. (2003)
China	Chinese fir	12.0	2067	57.90	68.29	157.71	Sun et al. (2003)
China	Chinese fir	-	-	-	25.57	-	Wang et al. (2004)
China	Chinese fir	24.0	2000	67.08	60.71	127.79	Niu et al. (2009)
China	Chinese fir	16.0	2000	95.70	43.47	139.17	Wang et al. (2009a)
China	Chinese fir	24.0	-	67.90	41.71	109.61	Wang et al. (2009b)
China	Chinese fir	28.0	-	71.10	46.51	117.61	Wang et al. (2009b)
China	Chinese fir	50.0	-	73.30	55.31	128.61	Wang et al. (2009b)
China	Chinese fir	40.0	1800	-	59.61	-	Wang et al. (2011)
China	Chinese fir	22.0	1135	-	46.22	-	Xiang et al. (2009)
China	Chinese fir	41.0	3000	145.00	83.00	232.00	Yang et al. (2005a)
China	Chinese fir	-	-	-	55.69	-	Yang et al. (2005b)
China	Chinese fir	33.0	1117	-	92.34	-	Yang et al. (2009)
China	Chinese fir	23.0	2500	-	54.58	-	Yang et al. (2010)
China	Chinese fir	6.0	3150	30.00	11.55	29.83	Yu et al. (2000)
China	Chinese fir	6.0	3050	12.30	34.08	36.16	Yu et al. (2000)
China	Chinese fir	6.0	3200	23.10	27.98	40.46	Yu et al. (2000)
China	Chinese fir	12.0	2625	29.40	23.29	41.75	Yu et al. (2000)
China	Chinese fir	6.0	3090	28.20	29.34	47.01	Yu et al. (2000)
China	Chinese fir	12.0	2950	61.40	8.19	57.65	Yu et al. (2000)
China	Chinese fir	12.0	2850	67.70	9.02	64.83	Yu et al. (2000)
China	Chinese fir	12.0	2940	65.70	15.51	69.76	Yu et al. (2000)

Country	Species	Age	Tree density	Above. C	Below. C	Ecosys. C	Reference
China	Chinese fir	6.0	3150	15.20	64.61	71.63	Yu et al. (2000)
China	Chinese fir	12.0	2530	19.70	73.20	85.29	Yu et al. (2000)
China	Chinese fir	12.0	2760	74.40	25.59	89.21	Yu et al. (2000)
China	Chinese fir	31.0	1550	102.90	25.00	117.08	Yu et al. (2000)
China	Chinese fir	31.0	1620	77.10	61.77	130.50	Yu et al. (2000)
China	Chinese fir	31.0	1095	110.80	42.55	143.70	Yu et al. (2000)
China	Chinese fir	31.0	1650	128.90	27.34	145.57	Yu et al. (2000)
China	Chinese fir	31.0	1200	130.80	34.41	155.02	Yu et al. (2000)
China	Chinese fir	23.0	2000	-	6.21	-	Zhang et al. (2009a)
China	Chinese fir	17.0	1400	-	63.89	-	Zhang et al. (2009b)
China	Chinese fir	17.0	1400	-	60.38	-	Zhang et al. (2009b)
China	Chinese fir	17.0	1400	-	60.34	-	Zhang et al. (2009b)
China	Chinese fir	17.0	1400	-	59.34	-	Zhang et al. (2009b)
China	Chinese fir	17.0	1400	-	64.34	-	Zhang et al. (2009b)
China	Chinese fir	14.0	2967	16.70	32.47	74.47	Zheng et al. (2008)
China	Chinese fir	7.0	-	14.67	62.42	77.66	Wang et al. (in press)
China	Chinese fir	22.0	-	59.14	78.77	137.91	Wang et al. (in press)
China	Chinese fir	14.0	-	45.93	-	171.40	Wang et al. (in press)
China	Chinese fir	-	-	85.98	67.10	153.08	Wang et al. (in press)
China	Chinese fir	88.0	-	156.71	101.65	258.36	Wang et al. (in press)
China	Chinese fir	19.0	2295	-	-	-	Zhou et al. (1992)
China	Chinese fir	21.0	1995	-	-	-	Zhou et al. (1992)
China	Chinese fir	16.0	1995	-	-	-	Zhou et al. (1992)
China	Chinese fir	16.0	1800	-	-	-	Zhou et al. (1992)
China	Chinese fir	23.0	1905	-	-	-	Zhou et al. (1992)
China	Chinese fir	-	1100	73.70	-	-	Cai (2009)
China	Chinese fir	-	-	-	73.47	187.57	Chen et al. (2005)
China	Chinese fir	28.0	-	28.34	89.94	113.28	Zhang et al. (2010)
Taiwan	Chinese fir	34.5	-	99.50	-	-	88
China	*Fokienia hodginsii*	-	-	53.01	89.60	142.60	73
China	Mason pine	10.0	700	68.90	49.57	124.97	Fang and Mo (2002)
China	Mason pine	33.0	1188	73.13	59.83	132.96	Fang et al. (2003)
China	Mason pine	50.0	850	113.23	81.26	194.49	Fang et al. (2003)
China	Mason pine	39.0	1207	105.39	96.52	201.91	Fang et al. (2003)
China	Mason pine	14.0	2100	73.94	9.16	83.10	Huang et al. (2011)
China	Mason pine	25.0	404	-	68.26	-	Wang et al. (in press)
China	Mason pine	-	-	-	46.75	-	Yang et al. (in press)
China	Mason pine	28.0	-	38.78	113.05	151.83	Zhang et al. (2010)
China	Mason pine	20.0	-	40.09	81.64	123.34	Wang et al. (in press)
China	Mason pine	30.0	-	55.75	95.81	154.41	Wang et al. (in press)
China	Mixed Chinese fir - broadleaf	20.0	2000	73.50	122.17	195.67	Huang et al. (2005)
China	Mixed Chinese fir - broadleaf	20.0	2000	73.25	122.17	195.42	Niu et al. (2009)

Country	Species	Age	Tree density	Above. C	Below. C	Ecosys. C	Reference
China	Mixed Chinese fir - broadleaf	24.0	2000	73.25	71.06	144.31	Wang et al. (2007b)
China	Mixed Chinese fir - broadleaf	16.0	2000	53.56	48.31	101.87	Wang et al. (2009a)
China	Mixed Chinese fir - broadleaf	16.0	2000	61.10	51.47	112.57	Wang et al. (2009a)
China	Mixed Chinese fir - broadleaf	28.0	-	28.35	130.24	158.59	Zhang et al. (2010)
China	Mixed Chinese fir - broadleaf	22.0	-	48.71	86.79	135.50	Wang et al. (in press)
China	Mixed Chinese fir - broadleaf	27.0	-	105.88	87.16	192.97	Wang et al. (in press)
China	Mixed *Cupresus* sp. - *Alnus* sp.	33.0	-	133.49	73.29	206.78	Wang et al. (in press)
Costa Rica	*Pinus caribaea*	17.0	1200	42.85	-	-	Arias et al. (2011)
Puerto Rico	*Pinus caribaea*	12.0	1450	47.00	51.27	98.27	Cuevas et al. (1991)
Sri Lanka	*Pinus caribaea*	27.0	-	103.00	-	-	Subasinghe and Munasinghe (2011)
Colombia	*Pinus patula*	8.0	1412	72.44	158.28	230.71	Loaiza Usaga et al. (2010)
Colombia	*Pinus patula*	21.5	936	121.59	162.64	284.22	Loaiza Usaga et al. (2010)
Colombia	*Pinus patula*	13.5	1174	129.49	162.34	291.83	Loaiza Usaga et al. (2010)
South Africa	*Pinus patula*	25.0	-	62.60	-	-	Christie and Scholes (1995)
South Africa	*Pinus patula*	15.0	-	59.20	-	-	Christie and Scholes (1995)
China	Slash pine	14.0	2100	27.00	41.47	73.47	Zheng et al. (2008)
USA (Florida)	Slash pine	12.0	2084	42.40	14.10	56.50	Clark et al. (2004)
USA (Florida)	Slash pine	0.0	0	0.50	60.10	60.60	Clark et al. (2004)
USA (Florida)	Slash pine	25.0	1301	67.70	39.30	108.70	Clark et al. (2004)
USA (Southern)	Slash pine	25.0	1800	113.56	52.30	165.86	Bracho et al. (2012)

5. References for Supplementary Information

Arias, D., Calvo-Alvarado, J., Richter, D.deB., Dohrenbusch, A. Productivity, aboveground biomass, nutrient uptake and carbon content in fast-growing tree plantations of native and inroduced spcies in the Southern Region of Costa Rica. Biomass Bioner. 35: 1779-1788 (2011).

Aye, Y.Y., Lee, D.K., Park, Y.., Park, G.E. Carbon stoage of 15-yearold Xylia xylocarpa and Pterocarpus macrocarpus plantations in the Katha District of Myanmar. For. Sci. Tech. 7: 134-140 (2011).

Bi, J. et al. Yield decline in Chinese Fir plantations: A simulation investigation with implications for model complexity. Can. J. For. Res. 37, 1615-1630 (2007).

Blanco, J.A., González, E. Exploring the sustainability of current management prescriptions for Pinus caribaea plantations in Cuba: a modelling approach. J. Trop. For. Sci. 22, 139-154 (2010).

Blanco, J.A., Seely, B., Welham, C., Kimmins, J.P., Seebacher, T.M. Testing the performance of FORECAST, a forest ecosystem model, against 29 years of field data in a Pseudotsuga menziesii plantation. Can. J. For. Res. 37, 1808-1820 (2007).

Bracho, R., Starr, G., Gholz, H.L., Martin, T.A., Cropper, W.P., Loescher, H.W. Controls on carbon dynamics by ecosystem structure and climate for southeastern U.S. slash pine plantations. Ecol. Monogra. 82: 101-128 (2012).

Cai S. Carbon storage and allocation in 26 an old Phobe bournei and Cunninghamia lanceolata plantations. J. Fujian For. Sci. Technol. 36, 9-25 (2009) (in Chinese).

Chen C. et al. Management effect of artificial forest of Phoebe bournie in Fujian. J. Fujian College For. 27, 101-104 (2007) (in Chinese).

Chen, G.S., Yang, Y.S., Wang, X.G., Xie, J.S., Gao, R., Li, Z. Root respiration in a natural forest and two plantations in subtropical China: seasonal dynamics and controlling factors. Acta Ecol. Sinica 25, 1941-1947 (2005) (in Chinese).

Chen G.-S., Yang, Z.-J., Gao, R., Xie, J.S., Guo, J.F., Huang, Z.-Q., Yang, Y.-S. Carbon storage in chronosequence of Chinese fir plantations in southern China. For. Ecol. Manage. (in press). DOI: 10.1016/j.foreco.2012.07.046.

Christie, S.I., Scholes, R.J. Carbon storage in eucalyptus and pine plantations in South Africa. Envir. Monit. Asses. 38: 231-241 (1995).

Clark, K.L., Gholz, H.L., Castro, M.S. Carbon dynamics along a chronosequence of slash pine plantations in north Florida. Ecol. Appl. 14: 1154-1171 (2004).

Cuevas, E., Brown, S., Lugo, A.E. Above- and belowground organic matter storage and production in a tropical pine plantation an a paired broadleaf secondary forest, Plant Soil, 135: 257-268 (1991).

Demessie, A., Sing, B.R., Lal, R. Soil carbon and nitrogen stocks under plantations in Gambo district, southern Ethiopia. J. Sustainable Forestry 30, 496-517 (2011).

De Ridder, M., Hubau, W., Van Den Bulcke, J., Van acker, J., Beeckman, H. The potential of plantations of Terminalia superba Engl. & Diels for wood and biomass production (Mayombe Forest, Democratic Republic of Congo). Ann. For. Scie. 67: 501 (2010).

Derwish, S., Schwentenmann, L., Olschewski, R., Hölscher, D. Estimation and economic evaluation of aboveground crbon storage of Tectona grandis plantations in Western Panama. New Forests 37: 227-240 (2009).

Du, G., Hong, L., Chen, F., Yang, Y., Dai, C.. Studies on successive rotation of Chinese fir. Forestry Tech. Dev. 15, 11–13 (2001) (in Chinese).

Fang, X., Tian, D., Xiang, W. Effects of different management regimes for cutover areas on soil carbon storage in Chinese fir plantations. Front. For. China 1, 38-42 (2006).

Fang, X., Tian, D., Xiang, W. Density, storage and distribution of carbon in Chiense fir plantations at fast growing stage. Scientia Silvae Sinicae 38, 14-19 (2002) (in Chinese).

Fang, Y.T., Mo, J.M. Study on carbon distribution and storage of a pine forest ecosystem in Dinghushan Biosphere Reserve. Guihaia 22: 305-310 (2002).

Fang, Y.T., Mo, J.M., Huang, Z.L., Ouyang, X.J. Carbon accumulation and distribution in Pinus massoniana and China superba mixed forest ecosystem in Dinghushan Biosphere Reserve. J. Trop. Subtrop. Bot. 11: 47-52 (2003).

Fang, S., Xue, J., Tang, L. Biomass production and carbon sequestration potential in poplar plantations with different management patterns. J. Environ. Manage. 85: 672-679 (2007).

Fonseca, W., Alice, F.E., Rey-Benayas, J.M. Carbon accumulation in aboveground an belowground biomass and soil of different age native forest plantations in the humid tropical lowlands of Costa Rica. New Forests 43: 197-211 (2012).

Guo, J.F., Yang, Y.S., Chen, G.S., Xie, J.S., Lin, P. Soil C and N pools in Chinese fir and evergreen forests and their changes with slash burning in mid-subtropical china. Pedosphere 16, 56-63 (2006).

He, Z., Yu, X. Study on problems of soil degradation in continuous cropping Chinese fir plantation. In Research on Site Degradation of Timber Plantation (eds. Sheng, W., Chen, B., Xu, H., Xu, X.) 243-250 (Chinese Science & Technology Press, Beijing, 1992) (in Chinese).

He, Y., Wang, Q., Wang, S., Yu, X. Characteristics of carbon and nitrogen of soil microbial biomass and their relationships with soil nutrients in Cunninghamia lanceolata plantations. Front. For. China 2, 266-271 (2007).

Hu, Y.L., Zeng, D.H., Fan, Z.P., Chen, G.S., Zhao, Q., Pepper, D. Changes in ecosystem carbon stocks following grassland afforestation of semiarid sandy soil in the southeastern Keerqin Sandy Lands, China. J. Arid Environ. 72: 2193-2200 (2008).

Huang, X.S., Wu, C.S., Hong, W., Li, Z.K., Cheng, Z.P. The relationship between stand density and biomass of two rotation Chinese fir plantations. J. Fujian College Forestry 31: 102-105 (2011).

Huang, Y. et al. C and N stocks under three plantation forest ecosystems of Chinese-fir, Michelia macclurei and their mixture. Acta Ecologica Sinica 25, 3146-3154 (2005) (in Chinese).

Huang, Y. et al. Changes in soil quality due to introduction of broad-leaf trees into clear-felled Chinese fir forest in the mid-subtropics of China. Soil Use Manage. 20, 418-425 (2004).

Huang Z., He, Z., Wan, X., Hu, Z., Fan, S., Yang, Y. Harvest residue management effects on tree growth and ecosystem carbon in a Chinese fir plantation in subtropical China. Plant Soil (in press). DOI 10.1007/s11104-012-1341-1.

Jiang, X. et al. 2009. A study on the growth characteristics of Phoebe bournei in natural forest and plantation stands. Acta Agriculturae Universitatis Jiangxiensis 31,1049-1054 (2009) (in Chinese).

Jiang, P., Meng, C., Zhou, G., Xu, Q. Comparative study of carbon storage in different forest stands in subtropical China. Botan. Rev. 77, 242-251 (2011).

Jiang,Y., Wang, B., Wang, Y., Yang, Q. Soil respiration in subtropical forests and model simulation of its relationships with soil temperature and moisture content. Chinese J. Appl. Ecol. 21, 1641-1648 (2010).

Kanowski, J., Catterall, C.P. Carbon stocks in above-ground biomass monoculture plantations, mixed species plantations and environmental restoration plantings in north-east Australia. Ecol. Manage. Restor. 11: 119-126 (2010).

Kaul, M., Mohren, G.M.J., Dahwal, V.K. Carbon storage and sequestration potential of selected tree species in India. Mitig. Adapt. Strateg. Glob. Change 15, 489-510. (2010).

Kimmins, J.P., Mailly, D., Seely, B. Modelling forest ecosystem net primary production: the hybrid simulation approach used in FORECAST. *Ecol. Model. 122*, 195-224 (1999).

Kimmins, J.P., Blanco, J.A., Seely, B., Welham, C., Scoullar, K. Forecasting Forest Futures: A Hybrid Modelling Approach to the Assessment of Sustainability of Forest Ecosystems and their Values. (Earthscan, London, 2010).

Kongsager, R., Napier, J., Mertz, O. The carbon sequestration potential of tree crop plantations. Mitig. Adapt. Strateg. Glob. Change (in press) DOI:10.1007/s11027-012-9417-z.

Kraenzel, M., Castillo, A., Moore, T., Potvin, C. Carbon storage of harvest-age teak (Tectona grandis) plantations, Panama. For. Ecol. Manage. 173: 213-225 (2003).

Li, S. Comprehensive evaluations of various afforestation patterns Phoebe bournei. Acta Agriculturae Universitatis Jiangxiensis 25, 100-103 (2003) (in Chinese).

Liao, H. et al. Planting density management to Cinnamoum camphora and Phoebe bournei. J. Fujian College For. 9, 80-84 (1989) (in Chinese).

Lin, X., Hong, L., Du, G., Preliminary study on soil quality evaluation on continuous cropping Chinese fir plantation. In Research on Site Degradation of Timber Plantation (eds. Sheng, W., Chen, B., Xu, H., Xu, X.) 267-275 (Chinese Science & Technology Press, Beijing, 1992) (in Chinese).

Loaiza Usuga, J.C., Rodríguez Toro, J.A., Ramírez Alzate, M.L., Lema Tapias, A.J. Estimation of biomass and carbón stocks in plants, soil an forest floor in different tropical forests. For. Ecol. Manage. 260: 1906-1913 (2010).

Long, H. et al. An analysis of the growth of Phoebe zhennan in Sichuan. J. Sichuan For. Sci. Technol. 32, 89-91 (2011) (in Chinese).

Luan. J. et al. Assessments of the impacts of Chinese fir plantation and natural regenerated forest on soil organic matter quality at Longmen mountain, Sichuan, China. Geoderma 156, 228-236 (2010).

Ma, X. et al. Effects of multirotation of Chinese fir plantations on granite sites. J. Fujian Col. Forestry 22, 289–294 (2002) (in Chinese).

Ma, M., Jiang H., Liu Y. Biomass, carbon content, carbon storage and their vertical distribution of Phoebe bournei artificial stand . Scientia Silvae Sinicae 44, 34-39 (2008) (in Chinese).

Ma, M., Luo, C., Jiang, H., Liu, Y., Li, X. 2009. Carbon sink in Phoebe bournei artificial forest ecosystem. Front. For. China 4: 140-145 (2009).

Mayer, D.G., Butler, D.G. Statistical validation. Ecol. Model. 68, 21-32 (1993).

Niu. D., Wang. S., Ouyang. Z. Comparisons of carbon storages in Cunninghamia lanceolata and Michelia macclurei plantations during a 22-year period in southern China. J. Environ. Sci. 21, 801-805 (2009).

Ordoñez et al. Carbon content in vegetation, litter, and soil under 10 different land-use and land-cover classes in the Central Highlands of Michoacan, Mexico. For. Ecol. Manage. 255: 2074-2084 (2008).

Peng, L. Biomass and productivity in 35 year old Phoebe bournei plantation. J. Fujian College For. 23, 128-131 (2003) (in Chinese).

Peng, L. Preliminary study on the biomass of Phoebe bournei plantation with different density and site condition. J. Fujian For. Sci. Technol. 35, 15-23 (2008) (in Chinese).

Peng, L. Study on nutrient elements of Phoebe bournei plantation in the different site conditions. J. Fujian For. Sci. Technol. 35, 10-15 (2008) (in Chinese).

Potvin, C., Whidden, E., Moore, T. A case study of carbon pools under three ifferent land-uses in Panamá. Climatic Change 67: 291-307 (2004).

Power, M., The predictive validation of ecological and environment models. Ecol. Model. 68, 33-50 (1993).

Razakamanarivo, R.H., Grinand, C., Razafindrakoto, M.A., Bernous, M., Albrecht, A. Mapping organic carbon stocks in eucaliptus plantations of the central highlands of Madagascar: a multiple regression approach. Geoderma 162: 335-346 (2011).

Redondo-Brenes, A. Growth, carbon sequestration, and management of native tree plantations in humid regions of Costa Rica. New Forests 34: 253-268 (2007).

Rios-Carrasco, E.d., Hoogh, R.d., Návar, J. Projection of carbón stocks in sites reforested with pinyon pine species in Northeastern Mexico. Arid Land Res. Manage. 23: 342-358 (2009).

Rong, Y. et al. Biomass productivity of the second generation Cunninghamia lanceolate plantation in northwest of Guangxi J. Guanxi Agricultural and Biological Science, 4. (2008) (in Chinese).

Ruiz-Jaen, M., Potvin, C. Can we predict carbon stocks in tropical ecosystems from tree diversity? Comparing species and functional diversity in a plantation and a natural forest. New Phytol. 189: 978-987 (2011).

Sang, P.M., Lamb, D., Bonner, M., Schmit, S. Carbon sequestration and soil fertility of tropical plantations and secondary forest established on degraded land. Plant Soil 362, 187-200 (2013).

Seely, B., Hawkins, C., Blanco, J.A., Welham, C., Kimmins, J.P. Evaluation of a mechanistic approach to mixedwood modelling. For. Chron. 84, 181-193 (2008).

Shao, Y. et al. Tests of soil organic carbon density modeled by InTEC in China's forest ecosystems. J. Environ. Manage. 85, 696-701 (2007).

Silver, W.L., Kueppers, L.M., Lugo, A.E., Osertag, R., Matzek, V. Carbon sequestration and plant community dynamics following reforestation of tropical pasture. Ecol. App. 14: 1115-1127 (2004).

Sing, P., Lohiyal, L.S. Biomass and carbon allocation in 8-year-ol Poplar (Populus deltoides Marsh) plantation in Tarai Agroforestry Systems of Central Himalaya, India. New York Sci. J. 2: 49-53 (2009).

Smith, P., et al. A comparison of the performance of nine soil organic matter models using datasets from seven long-term experiments. Geoderma 81, 153-225 (1997).

Subasinghe, S.M.C.U.P., Munasinghe, G.B. Estimation of above ground tree biomass an carbon of Pinus caribaea (Morelet). J. Trop. For. Environ. 1: 56-70 (2011).

Sun, X. Study on the effect of thinning on the growth of mixed forest of Phoebe bournei and Cunninghamia lanceolata. Subtropical Agriculture Res. 4, 184-187 (2008) (in Chinese).

Sun, Q., Yang, C., Jiao, R. The changes of soil properties of the successive Chinese fir plantation in Dagang Mountain of Jiangxi Province. Scientia Silvae Sinicae 39, 1–5 (2003).

Theil, H. Applied Econometric Forecasting (North-Holland, Amsterdam, 1966).

Tian, D.L. Chinese Fir Forest Ecology (Science Press, Beijing, 2003).

Tong, G. Study on the effect of aspect and afforestation density on Machilus Paunoi Kanenira young forest growth. J. Fujian For. Sci. Technol. 37, 70-72 (2010) (in Chinese).

Vanclay, J.K., Skovsgaard, J.P. Evaluating forest growth models. Ecol. Model. 98, 1-12 (1997).

Wang, W. et al. Evaluation of the effects of forest management strategies on carbon sequestration in evergreen broad-leaved (Phoebe bournei) plantation forests using FORECAST ecosystem model. For. Ecol. Manage. DOI 10.1016/j.foreco.2012.06.044 (2012).

Wang et al. Effects of tree species mixture on soil organic carbon stocks and greenhouse gas fluxes in subtropical plantations in China. For. Ecol. Manage. (in press) DOI: 10.1016/j.foreco.2012.04.005.

Wang, F.E. et al. Microbial biomass carbon, nitrogen and phosphorus in the soil profiles of different vegetation covers established for soil rehabilitation in a red soil region of southeastern China. Nutrient Cycling Agroecosys. 68, 181-189 (2004).

Wang Q, Wang S, Yu X, Zhang J, Liu Y. Soil carbon mineralization potential and its effect on soil active organic carbon in evergreen broadleaved forest and Chinese fir plantation. Chinese J. Ecol. 26, 1918-1923 (2007b) (in Chinese).

Wang, D., Wang, B., Dai, W., Li, P., Hu, W., Guo, H. The variation characteristics of soil organic carbon and its influence factor in different developing stages of Chinese fir plantations. For. Res. 22: 667-671. 2009

Wang, Q., Wang, S., Zhang, J. Assessing the effects of vegetation types on carbon storage fifteen years after reforestation on a Chinese fir site. For. Ecol. Manage. 258, 1437-1441 (2009a).

Wang C, Huang Q, Yang Z, Huang R, Chen G. Analysis of vertical profiles of soil CO2 efflux in Chinese fir plantation. Acta Ecol. Sinica 31, 5711-5719 (2011).

Watanabe, Y., Msunaga, T., Owusu-sekyere, E., Buri, M.M., Olaele, O.I., Wakatsuki, T. Evaluation of growth and carbon storage as influenced by soil chemical properties and moisture on teak (Tectona grandis) in Ashanti region, Ghana. J. Food Agri. Environ. 7: 640-645 (2009).

Wauters, J.B., Coudert, S., Grallien, E., Jonard, M., Ponette, Q. Carbon stock in rubber tree plantations in Western Ghana and Mato Grosso (Brazil). For. Ecol. Manage. 255: 2347-2361 (2008).

Wei, X., Blanco, J.A., Jiang, H., Kimmins, J.P. Effects of nitrogen deposition on carbon sequestration in Chinese fir forests. Sci. Tot. Environ. 416, 351-361 (2012).

Wei, H., Ma, X. Study on the carbon storage and distribution of Phoebe bournei plantation ecosystem of different growing stages. Yantai Normal University J. (Nat. Sci.) 22, 130-133 (2006) (in Chinese).

Wu, W. Study on stand structure and growth law of near mature Phoebe bournei plantation. Develop. For. Sci. Technol. 23, 75-78 (2009) (in Chinese).

Xiang, W., Chai, H., Tian, D., Peng, C. Marginal effects of silvicultural treatments on soil nutrients following harvest in a Chinese fir plantation. Soil Sci. Plant Nutrition 55, 523-531 (2009).

Yang, F., Liu, J., Zhuang, D., Hu, Y. Soil nutrient changes under artificial coniferous forests in the red soil hilly region in southeast China. In Gao, W., Shaw, D.R. Remote Sensing and Modelling of Ecosystems for Sustainability II. Proceedings of SPIE Vol. 5884 (SPIE, Bellingham, WA, 2005) · 0277-786X/05/$15 · doi: 10.1117/12.614317).

Yang, Y.S. et al. Carbon and nitrogen pools in Chinese fir and evergreen broadleaved forests and changes associated with felling and burning in mid-subtropical China. For. Ecol. Manage. 216, 216-226 (2005a).

Yang, Y.S., Chen, G.-S., Wang, X.G., Xie, J.S., Gao, R., Li, Z., Jin, Z. Response of soil CO2 to forest conversion in subtropical zone of China. Acta Ecol. Sinica 25: 1684-1690 (2005b).

Yang, Y., Guo, J., Chen, G., Yin, Y., Gao, R., Lin, C. effects of forest conversion on soil labile organic carbon fractions and aggregate stability in subtropical China. Plant Soil 323: 153-162 (2009).

Yang, Z.J., Chen, G.S., Xie, J.S., Yang, Y.S. Litter fall production an carbon return in Cunningahmia lanceolata, Schina superba, and their mixed plantations. Chi. J. App. Ecol. 21: 2235-2240. (2010)

Yen, T.M., Lee, J.S. Comparing aboveground carbon sequestration between moso bamboo (Phyllostachys heterocycla) and China fir (Cunninghamia lanceolata) forests based on the allometric model. For. Ecol. Manage. 261, 995-1002 (2011).

Yu, Y. et al. Effects of continuous plantation of Chinese fir on soil physical properties. J. Nanjing For. Univ. 24, 36–40 (2000) (in Chinese).

Zhang, W., Wu, Z. Experiment study on different nurturing measures of Phoebe bournei plantation. J. Minxi Vocational and Technical College 9, 1-4. (2007) (in Chinese).

Zhang, J., Wang, S., Feng, Z., Wang, Q. Stability of soil organic carbon changes in successive rotations of Chinese fir (Cunninghamia lanceolata (Lamb.) Hook) plantations. J. Environ. Sci. 21, 352-359 (2009a).

Zhang, J., Wang, S.L., Feng, Z.W., Wang, Q.K. Carbon mineralization of soils from native evergreen broadleaf forest and three plantations in mid-subtropic China. Comm. Soil Sci. Plant Anal. 40, 1964-1982 (2009b).

Zhang, J. et al. Carbon storage and its sequestration potential by ecological service forest in Zhejiang. Acta Ecol. Sinica 30, 3839-2348 (2010).

Zheng, H. et al. Variation of carbon storage by different reforestation types in the hilly red soil region of southern China. For. Ecol. Manage. 255, 1113-1121 (2008).

Zheng, J.X., Liu, X.F., Gao, R., Yang, Z.J., Yang, Y.S. 2009. Carbon storage and allocation in the Phoebe bournei plantations in Namping, Fujian Province. J. Subtropical Resources and Environment 4, 59-65 (2009).

Zhou, X., Luo, R., Ye, J. Influence of continuous cropping on soil nutrients and their feedback effects. In Research on Site Degradation of Timber Plantation (eds. Sheng, W., Chen, B., Xu, H., Xu, X.) 100-107 (Chinese Science & Technology Press, Beijing, 1992) (in Chinese).
